# Supplementary material for: Structural variation of types IV-A1- and IV-A3-mediated CRISPR interference
Source: Nat Commun. 2024 Oct 29;15:9306. doi: 10.1038/s41467-024-53778-1 (PMC11519345; doi:10.1038/s41467-024-53778-1)

# Supplementary information for:

# Structural variation of types IV-A1- and IV-A3-mediated CRISPR interference

Čepaitė, R.[1], Klein, N.[2], Mikšys, A.[1,3], Camara-Wilpert, S.[4], Ragožius, V.[1], Benz, F.[5,6], Skorupskaitė A.[1], Becker, H.[2], Žvejytė, G.[1], Steube, N.[7], Hochberg, G.K.A.[7,8,9], Randau, L.[2,9], Pinilla-Redondo, R.[4], Malinauskaitė, L.[*1,10], Pausch, P.[*1]

[1] LSC-EMBL Partnership Institute for Genome Editing Technologies, Life Sciences Center, Vilnius University, Saulėtekio al. 7, 10257 Vilnius, Lithuania
[2] Department of Biology, Philipps-Universität Marburg, Hans-Meerwein-Straße 6, D-35032, Marburg, Germany
[3] Current: ATEM Structural Discovery GmbH, Büchelstraße 54, 42855 Remscheid, Germany
[4] Department of Biology, Section of Microbiology, University of Copenhagen, Universitetsparken 15, 2100 Copenhagen, Denmark
[5] Institut Pasteur, Université Paris Cité, CNRS UMR6047, 75015 Paris, Synthetic Biology, Paris 75015, France
[6] Institut Pasteur, Université Paris Cité, CNRS UMR3525, Microbial Evolutionary Genomics, Paris 75015, France
[7] Evolutionary Biochemistry Group, Max Planck Institute for Terrestrial Microbiology, Karl-von-Frisch-Straße 10, D-35043, Marburg, Germany
[8] Department of Chemistry, Philipps-Universität Marburg, Hans-Meerwein-Straße 4, D-35032, Marburg, Germany
[9] Center for Synthetic Microbiology (SYNMIKRO), Karl-von-Frisch-Straße 14, 35043 Marburg, Germany
[10] Current: BioNTech UK Ltd, Cambridge Biomedical Campus, Francis Crick Ave, Trumpington, Cambridge CB2 0QH, UK

Correspondence and requests for materials should be addressed to L.M. (email: lina.malinauskaite@biontech.co.uk) and P.P. (email: patrick.pausch@gmc.vu.lt).

## Content:

**Supplementary Tables 1-4** (p. 2-10)

**Supplementary Figures 1-21** (p. 11-34)

**Supplementary References** (p. 35)

**Supplementary Source Data** (p. 36-61)

## SUPPLEMENTARY TABLES

**Supplementary Tab. 1 | Cryo-EM data collection, refinement and validation statistics for type IV-A1 in absence and presence of DinG, and type IV-A3 in absence of DinG.**

| | **Type IV-A1 in complex with DNA (EMD-19046, EMD-19120, EMD-19124, PDB-ID: 8RC3)** | **Type IV-A1 in complex with DNA and DinG (EMD-19125, EMD-19126, EMD-19127, EMD-51026, PDB-ID: 8REJ)** | **Type IV-A3 in complex with DNA (EMD-19045, PDB-ID: 8RC2)** |
|---|---|---|---|
| **Data collection and processing** | | | |
| Magnification | ×92000 | | ×92000 |
| Voltage (kV) | 200 | | 200 |
| Exposure ($e^{-}/Å^2$) | 30 | | 30 |
| Defocus range (μm) | -2.0 to -1.0 | | -2.0 to -1.0 |
| Pixel size (Å) | 1.1 | | 1.1 |
| Initial particles | 1,022,768 | | 1,082,094 |
| Final particles | 103,699 | 55,924 | 104,035 |
| Symmetry imposed | C1 | C1 | C1 |
| Resolution (Å) | 2.96 | 3.18 | 3.1 |
| FSC cutoff | 0.143 | 0.143 | 0.143 |
| Map resolution range (Å) | 2.5-7 | 3-8 | 2.5-7 |
| **Refinement** | | | |
| Model resolution (Å) | 3.1 | 3.7 | 3.3 |
| 0.143 FSC Threshold (Å) | 2.0 | 2.2 | 2.8 |
| Map sharpening *B* factor | 96.7 | 81.3 | 70 |
| Model composition | | | |
| Non-hydrogen atoms | 19897 | 24687 | 19755 |
| Protein residues | 2297 | 2877 | 2304 |
| Nucleotides | 112 | 129 | 94 |
| Ligands | ZN: 1 | ZN: 1 | ZN: 1 |
| *B* factors ($Å^2$) | | | |
| Protein | 67.55 | 113.57 | 124.34 |
| Nucleotide | 102.27 | 125.16 | 132.81 |
| Ligand | 92.18 | 104.23 | 166.56 |
| **Validation** | | | |
| Bonds (r.m.s. deviations) | | | |
| Length (Å) (# > 4s) | 0.004 (0) | 0.003 (0) | 0.006 (0) |
| Angles (°) (# > 4s) | 0.556 (3) | 0.544 (1) | 0.614 (0) |
| MolProbity score | 1.59 | 1.57 | 1.41 |
| Clash score | 10.06 | 10.20 | 7.49 |
| Poor rotamers (%) | 0 | 0 | 0.42 |
| Ramachandran plot (%) | | | |

| | | | |
|---|---|---|---|
| Outliers | 0.00 | 0.00 | 0.00 |
| Allowed | 2.29 | 2.08 | 1.32 |
| Favored | 97.71 | 97.92 | 98.68 |
| Rama-Z score, whole (r.m.s. Rama-Z) | 0.49 (0.17) | 0.80 (0.16) | 0.85 (0.17) |
| CC (mask) | 0.86 | 0.81 | 0.82 |
| CC (box) | 0.86 | 0.89 | 0.73 |

**Supplementary Tab. 2 | Cryo-EM data collection, refinement and validation statistics for type IV-A3 in presence of DinG.**

| | **Type IV-A3 in complex with DNA and DinG (state I) (EMD-19688, PDB-ID: 8S35)** | **Type IV-A3 in complex with DNA and DinG (state II) (EMD-19689, PDB-ID: 8S36)** | **Type IV-A3 in complex with DNA and DinG (state III) (EMD-19690, PDB-ID: 8S37)** |
|---|---|---|---|
| **Data collection and processing** | | | |
| Magnification | ×92000 | | |
| Voltage (kV) | 200 | | |
| Exposure ($e^{-}/Å^2$) | 30.31 | | |
| Defocus range (μm) | -2.0 to -1.0 | | |
| Pixel size (Å) | 1.1 | | |
| Initial particles | 4,069,893 | | |
| Final particles | 45,309 | 48,197 | 46,284 |
| Symmetry imposed | C1 | C1 | C1 |
| Resolution (Å) | 2.90 | 2.88 | 2.89 |
| FSC cutoff | 0.143 | 0.143 | 0.143 |
| Map resolution range (Å) | 2.5-7 | 2.5-7 | 2.5-7 |
| **Refinement** | | | |
| Model resolution (Å) | 3.6 | 3.7 | 3.6 |
| 0.143 FSC Threshold (Å) | 3.0 | 3.0 | 3.0 |
| Map sharpening *B* factor | 71.1 | 72.6 | 71.9 |
| Model composition | | | |
| Non-hydrogen atoms | 25082 | 24249 | 25085 |
| Protein residues | 2936 | 2838 | 2947 |
| Nucleotides | 112 | 108 | 107 |
| Ligands | ZN: 1 | ZN: 1 | ZN: 1 |
| *B* factors ($Å^2$) | | | |
| Protein | 204.28 | 203.73 | 221.25 |
| Nucleotide | 198.85 | 204.32 | 205.08 |
| Ligand | 215.96 | 217.23 | 192.95 |
| **Validation** | | | |
| Bonds (r.m.s. deviations) | | | |
| Length (Å) (# > 4s) | 0.004 (0) | 0.004 (0) | 0.004 (0) |
| Angles (°) (# > 4s) | 0.726 (10) | 0.728 (7) | 0.659 (1) |

| | | | |
|---|---|---|---|
| MolProbity score | 1.51 | 1.52 | 1.48 |
| Clash score | 9.69 | 10.07 | 9.09 |
| Poor rotamers (%) | 0.41 | 0.81 | 0.90 |
| Ramachandran plot (%) | | | |
| Outliers | 0.00 | 0.07 | 0.14 |
| Allowed | 0.28 | 0.50 | 0.31 |
| Favored | 99.72 | 99.43 | 99.55 |
| Rama-Z score, whole (r.m.s. Rama-Z) | -0.29 (0.15) | -1.16 (0.15) | -1.05 (0.15) |
| CC (mask) | 0.82 | 0.81 | 0.82 |
| CC (box) | 0.90 | 0.89 | 0.89 |

## Supplementary Tab. 3 | DNA oligonucleotides.

| **Sequence name** | **Purpose** | **Sequence (5‘ → 3‘)** |
|---|---|---|
| Type IV-A1 target strand | cryo-EM sample reconstitution | HO-CGGTCGGGTCATACGTCGCGTCTCGAATCTGATGCGTAACTTGGATGCTTCGTGCGTGATG |
| Type IV-A1 non-target strand | cryo-EM sample reconstitution | HO-CATCACGCACGAAGACCAAGTCAATGCTTAGTCTAATACCTGCGCTCGTATGACCCGACCG |
| Type IV-A3 target strand | cryo-EM sample reconstitution | HO-CCCTCCCTCCAGCTTCCGAGACCCTTCGGGAGGTGCATCCCGGTCTCGCTTGGCCTCCTC |
| Type IV-A3 non-target strand | cryo-EM sample reconstitution | HO-GAGGAGGCCAAGATCTCAATTTCGTACAAGAAATCCTTTGAGATGAAGCTGGAGGGAGGG |
| EMSA probe forward primer | EMSA probe amplification | CTATCCCATATCACCAGCTCAC |
| EMSA probe reverse primer | EMSA probe amplification | ATTO647N-GTTGATCGGCACGTAAGAG |

## Supplementary Tab. 4 | Plasmids.*

| **ID** | **Purpose** | **Features** | **Selection marker** |
|---|---|---|---|
| pRC002 Ref. [1] | Protein purification | pRSF-Duet1 derived plasmid containing type IV-A3 genes with C-terminally hexa-histidine tagged *csf2* (*cas7*) | Kanamycin |
| pRC004 Ref. [1] | Protein purification | pYTK095 derived plasmid containing a repeat-spacer-repeat motif from type IV-A3 CRISPR-array. Contains non-targeting BsaI-GG stuffer spacer. | Ampicillin |
| pNK25 | Protein purification | pRSF-Duet1 derived plasmid containing type IV-A1 genes with C-terminally hexa-histidine tagged *csf1* (*cas8*) in MCS1 and *dinG* in MCS2 | Kanamycin |
| pAG33 | Protein purification | pUC19 derived plasmid containing a repeat-spacer-repeat motif from type IV-A1 CRISPR-array. | Ampicillin |
| pNK27 | Protein purification | pRSF-Duet1 derived plasmid containing type IV-A1 genes with C-terminally hexa-histidine tagged *csf1* (*cas8*) in MCS1 | Kanamycin |

| | | | |
|---|---|---|---|
| pAG19 | Protein purification | pET20b derived plasmid with C-terminally hexa-histidine tagged *dinG* | Ampicillin |
| pSR77 | Repression assay | pRSFDuet-1 derived plasmid containing type IV-A1 genes and a repeat-spacer-repeat mini-CRISPR (non-targeting control) | Kanamycin |
| pSR102 | Repression assay | pRSFDuet-1 derived plasmid containing type IV-A1 genes and a repeat-spacer-repeat mini-CRISPR targeting *lacZ* (targeting control) | Kanamycin |
| pNK92 | Repression assay | pRSFDuet-1 derived plasmid encoding DinG with amino acid substitution R440A and a repeat-spacer-repeat motif targeting *lacZ* | Kanamycin |
| pNK93 | Repression assay | pRSFDuet-1 derived plasmid encoding DinG with amino acid substitution W494A and a repeat-spacer-repeat motif targeting *lacZ* | Kanamycin |
| pNK94 | Repression assay | pRSFDuet-1 derived plasmid encoding DinG with amino acid substitution K661A and a repeat-spacer-repeat motif targeting *lacZ* | Kanamycin |
| pNK95 | Repression assay | pRSFDuet-1 derived plasmid encoding DinG with amino acid substitution R493A and a repeat-spacer-repeat motif targeting *lacZ* | Kanamycin |
| pNK96 | Repression assay | pRSFDuet-1 derived plasmid encoding DinG with amino acid substitution Q390A and a repeat-spacer-repeat motif targeting *lacZ* | Kanamycin |
| pNK97 | Repression assay | pRSFDuet-1 derived plasmid encoding DinG with amino acid substitution K463A and a repeat-spacer-repeat motif targeting *lacZ* | Kanamycin |
| pNK98 | Repression assay | pRSFDuet-1 derived plasmid encoding DinG with amino acid substitution I567W and a repeat-spacer-repeat motif targeting *lacZ* | Kanamycin |
| pNK99 | Repression assay | pRSFDuet-1 derived plasmid encoding DinG with amino acid substitution R653A and a repeat-spacer-repeat motif targeting *lacZ* | Kanamycin |
| pNK100 | Protein purification | pET20b derived plasmid encoding DinG with amino acid substitution R440A | Ampicillin |
| pNK101 | Protein purification | pET20b derived plasmid encoding DinG with amino acid substitution W494A | Ampicillin |
| pNK102 | Protein purification | pET20b derived plasmid encoding DinG with amino acid substitution R653A | Ampicillin |
| pNK112 | Repression assay | pYTK095 derived plasmid containing a repeat-spacer-repeat mini-CRISPR from type IV-A3 CRISPR-array with a spacer targeting *lacZ*. | Ampicillin |
| pNK113 | Repression assay | pRSF-Duet1 derived plasmid containing type IV-A3 genes and encoding DinG with a truncated C-terminus (Δ617-624) | Kanamycin |
| pNK114 | Repression assay | pRSF-Duet1 derived plasmid containing type IV-A3 genes and encoding DinG with amino acid substitution Y527W | Kanamycin |
| pNK115 | Repression assay | pRSF-Duet1 derived plasmid containing type IV-A3 genes and encoding DinG with amino acid substitutions R537A, T538W and F539A | Kanamycin |
| pNK117 | Repression assay | pRSF-Duet1 derived plasmid containing type IV-A3 genes and encoding DinG with amino acid substitution L588W | Kanamycin |

| | | | |
|---|---|---|---|
| pNK118 | Repression assay | pRSF-Duet1 derived plasmid containing type IV-A3 genes and encoding DinG with amino acid substitution Y527A | Kanamycin |
| pNK119 | Repression assay | pRSF-Duet1 derived plasmid containing type IV-A3 genes and encoding DinG with amino acid substitution R544A | Kanamycin |
| pNK121 | Repression assay | pRSF-Duet1 derived plasmid containing type IV-A3 genes and encoding DinG with amino acid substitutions R605A and R608A | Kanamycin |
| pNK122 | Repression assay | pRSF-Duet1 derived plasmid containing type IV-A3 genes and encoding DinG with amino acid substitution K288A | Kanamycin |
| pNK125 | Repression assay | pRSF-Duet1 derived plasmid containing type IV-A3 genes and encoding DinG with amino acid substitution L588A | Kanamycin |
| pNK126 | Protein purification | pRSF-Duet1 derived plasmid encoding DinG with amino acid substitution Y527A | Kanamycin |
| pNK127 | Protein purification | pRSF-Duet1 derived plasmid encoding DinG with amino acid substitutions R537A, T538W and F539A | Kanamycin |
| pNK128 | Repression assay | pRSF-Duet1 derived plasmid containing type IV-A3 genes and encoding DinG with amino acid substitutions N412A, I418A and Q422A | Kanamycin |
| pSR24 | Mismatch tolerance assays in *E. coli* | pCDFDuet-1 derived plasmid containing a repeat-spacer-repeat mini-CRISPR from type IV-A1 CRISPR array with no mutations (no mismatch control) | Spectinomycin |
| pHB01 | Mismatch tolerance assays in *E. coli* | pCDFDuet-1 derived plasmid containing a repeat-spacer-repeat mini-CRISPR from type IV-A1 CRISPR array with spacer mutation in position 1 (C1G) | Spectinomycin |
| pHB02 | Mismatch tolerance assays in *E. coli* | pCDFDuet-1 derived plasmid containing a repeat-spacer-repeat mini-CRISPR from type IV-A1 CRISPR array with spacer mutations in positions 2,3,4 (A2T, T3A, C4G) | Spectinomycin |
| pHB03 | Mismatch tolerance assays in *E. coli* | pCDFDuet-1 derived plasmid containing a repeat-spacer-repeat mini-CRISPR from type IV-A1 CRISPR array with spacer mutation in position 5 (C5G) | Spectinomycin |
| pHB04 | Mismatch tolerance assays in *E. coli* | pCDFDuet-1 derived plasmid containing a repeat-spacer-repeat mini-CRISPR from type IV-A1 CRISPR array with spacer mutation in position 6 (A6G) | Spectinomycin |
| pHB05 | Mismatch tolerance assays in *E. coli* | pCDFDuet-1 derived plasmid containing a repeat-spacer-repeat mini-CRISPR from type IV-A1 CRISPR array with spacer mutation in position 7 (A7T) | Spectinomycin |
| pHB06 | Mismatch tolerance assays in *E. coli* | pCDFDuet-1 derived plasmid containing a repeat-spacer-repeat mini-CRISPR from type IV-A1 CRISPR array with spacer mutation in position 8 (G8C) | Spectinomycin |
| pNK50 | Mismatch tolerance assays in *E. coli* | pCDFDuet-1 derived plasmid containing a repeat-spacer-repeat mini-CRISPR from type IV-A1 CRISPR array with spacer mutation in position 4 (C4G) | Spectinomycin |
| pNK71 | Mismatch tolerance assays in *E. coli* | pCDFDuet-1 derived plasmid containing a repeat-spacer-repeat mini-CRISPR from type IV-A1 CRISPR array with spacer mutation in position 2 (A2T) | Spectinomycin |
| pNK72 | Mismatch tolerance assays in *E. coli* | pCDFDuet-1 derived plasmid containing a repeat-spacer-repeat mini-CRISPR from type IV-A1 CRISPR array with spacer mutation in position 3 (T3A) | Spectinomycin |

| | | | |
|---|---|---|---|
| pNK59 | Mismatch tolerance assays in *E. coli* | pCDFDuet-1 derived plasmid containing a repeat-spacer-repeat mini-CRISPR from type IV-A1 CRISPR array with spacer mutation in position 9 (T9A) | Spectinomycin |
| pNK60 | Mismatch tolerance assays in *E. coli* | pCDFDuet-1 derived plasmid containing a repeat-spacer-repeat mini-CRISPR from type IV-A1 CRISPR array with spacer mutation in position 10 (T10A) | Spectinomycin |
| pNK61 | Mismatch tolerance assays in *E. coli* | pCDFDuet-1 derived plasmid containing a repeat-spacer-repeat mini-CRISPR from type IV-A1 CRISPR array with spacer mutation in position 11 (A11T) | Spectinomycin |
| pNK62 | Mismatch tolerance assays in *E. coli* | pCDFDuet-1 derived plasmid containing a repeat-spacer-repeat mini-CRISPR from type IV-A1 CRISPR array with spacer mutation in position 1 (C12G) | Spectinomycin |
| pNK63 | Mismatch tolerance assays in *E. coli* | pCDFDuet-1 derived plasmid containing a repeat-spacer-repeat mini-CRISPR from type IV-A1 CRISPR array with spacer mutation in position 13 (G13C) | Spectinomycin |
| pNK64 | Mismatch tolerance assays in *E. coli* | pCDFDuet-1 derived plasmid containing a repeat-spacer-repeat mini-CRISPR from type IV-A1 CRISPR array with spacer mutation in position 14 (C14G) | Spectinomycin |
| pNK65 | Mismatch tolerance assays in *E. coli* | pCDFDuet-1 derived plasmid containing a repeat-spacer-repeat mini-CRISPR from type IV-A1 CRISPR array with spacer mutation in position 15 (A15T) | Spectinomycin |
| pNK66 | Mismatch tolerance assays in *E. coli* | pCDFDuet-1 derived plasmid containing a repeat-spacer-repeat mini-CRISPR from type IV-A1 CRISPR array with spacer mutation in position 16 (T16A) | Spectinomycin |
| pNK67 | Mismatch tolerance assays in *E. coli* | pCDFDuet-1 derived plasmid containing a repeat-spacer-repeat mini-CRISPR from type IV-A1 CRISPR array with spacer mutations in positions 17-20 (CAGA→GTCT) | Spectinomycin |
| pNK68 | Mismatch tolerance assays in *E. coli* | pCDFDuet-1 derived plasmid containing a repeat-spacer-repeat mini-CRISPR from type IV-A1 CRISPR array with spacer mutations in positions 21-24 (TTCG→AAGC) | Spectinomycin |
| pNK69 | Mismatch tolerance assays in *E. coli* | pCDFDuet-1 derived plasmid containing a repeat-spacer-repeat mini-CRISPR from type IV-A1 CRISPR array with spacer mutations in positions 25-28 (AGAC→TCTG) | Spectinomycin |
| pNK70 | Mismatch tolerance assays in *E. coli* | pCDFDuet-1 derived plasmid containing a repeat-spacer-repeat mini-CRISPR from type IV-A1 CRISPR array with spacer mutations in positions 29-32 (GCGA→CGCT) | Spectinomycin |
| pSR14 | Mismatch tolerance assays in *E. coli* | pACYCDuet-1 derived plasmid containing a type IV-A1 target sequence | Chloramphenicol |
| pSR15 | Mismatch tolerance assays in *E. coli* | pACYCDuet-1 derived plasmid containing a non-target sequence | Chloramphenicol |
| pHB07 | Mismatch tolerance assays in *E. coli* | pACYCDuet-1 derived plasmid containing a type IV-A1 protospacer with mutation in position 1 (G1C) | Chloramphenicol |
| pHB08 | Mismatch tolerance assays in *E. coli* | pACYCDuet-1 derived plasmid containing a type IV-A1 protospacer with mutation in position 2 (T2A) | Chloramphenicol |
| pHB09 | Mismatch tolerance assays in *E. coli* | pACYCDuet-1 derived plasmid containing a type IV-A1 protospacer with mutation in position 3 (A3T) | Chloramphenicol |

| | | | |
|---|---|---|---|
| pHB10 | Mismatch tolerance assays in *E. coli* | pACYCDuet-1 derived plasmid containing a type IV-A1 protospacer with mutation in position 4 (G4C) | Chloramphenicol |
| pHB11 | Mismatch tolerance assays in *E. coli* | pACYCDuet-1 derived plasmid containing a type IV-A1 protospacer with mutation in position 5 (G5C) | Chloramphenicol |
| pHB12 | Mismatch tolerance assays in *E. coli* | pACYCDuet-1 derived plasmid containing a type IV-A1 protospacer with mutation in position 6 (T6A) | Chloramphenicol |
| pHB13 | Mismatch tolerance assays in *E. coli* | pACYCDuet-1 derived plasmid containing a type IV-A1 protospacer with mutation in position 7 (T7A) | Chloramphenicol |
| pHB14 | Mismatch tolerance assays in *E. coli* | pACYCDuet-1 derived plasmid containing a type IV-A1 protospacer with mutation in position 8 (C8G) | Chloramphenicol |
| pHB15 | Mismatch tolerance assays in *E. coli* | pACYCDuet-1 derived plasmid containing a type IV-A1 protospacer with mutations in position 2,3,4 (T2A, A3T, G4C) | Chloramphenicol |
| pNK82 | Mismatch tolerance assays in *P. oleovorans* | pSR106 derived plasmid containing a repeat-spacer-repeat motif from type IV-A1 with a spacer mutation in position 1 (G1C) | Spectinomycin |
| pNK83 | Mismatch tolerance assays in *P. oleovorans* | pSR106 derived plasmid containing a repeat-spacer-repeat motif from type IV-A1 with a spacer mutation in position 2 (T2A) | Spectinomycin |
| pNK84 | Mismatch tolerance assays in *P. oleovorans* | pSR106 derived plasmid containing a repeat-spacer-repeat motif from type IV-A1 with a spacer mutation in position 3 (G3C) | Spectinomycin |
| pNK85 | Mismatch tolerance assays in *P. oleovorans* | pSR106 derived plasmid containing a repeat-spacer-repeat motif from type IV-A1 with a spacer mutation in position 4 (A4T) | Spectinomycin |
| pNK86 | Mismatch tolerance assays in *P. oleovorans* | pSR106 derived plasmid containing a repeat-spacer-repeat motif from type IV-A1 with a spacer mutation in position 5 (T5A) | Spectinomycin |
| pNK87 | Mismatch tolerance assays in *P. oleovorans* | pSR106 derived plasmid containing a repeat-spacer-repeat motif from type IV-A1 with a spacer mutation in position 6 (G6C) | Spectinomycin |
| pNK88 | Mismatch tolerance assays in *P. oleovorans* | pSR106 derived plasmid containing a repeat-spacer-repeat motif from type IV-A1 with a spacer mutation in position 7 (C7G) | Spectinomycin |
| pNK89 | Mismatch tolerance assays in *P. oleovorans* | pSR106 derived plasmid containing a repeat-spacer-repeat motif from type IV-A1 with a spacer mutation in position 8 (G8C) | Spectinomycin |
| pNK91 | Mismatch tolerance assays in *P. oleovorans* | pSR106 derived plasmid containing a repeat-spacer-repeat mini-CRISPR from type IV-A1 (no mismatch control) | Spectinomycin |
| pSR106 | Mismatch tolerance assays in *P. oleovorans* | pSEVA424 derived plasmid containing a repeat-spacer-repeat mini-CRISPR from type IV-A1 (non-targeting control) | Spectinomycin |
| pRC040 | Mismatch tolerance assays *in vitro* | pRSF-Duet1 derived plasmid containing type IV-A3 genes with C-terminally hexa-histidine tagged *csf2* (*cas7*) with a Δ191-208 (GSSG) mutation | Kanamycin |

| | | | |
|---|---|---|---|
| pRC041 | Mismatch tolerance assays *in vitro* | pRSF-Duet1 derived plasmid containing type IV-A3 genes with C-terminally hexa-histidine tagged *csf2* (*cas7*) with a Δ184-216 (GSSG) mutation | Kanamycin |
| pRC042 | Mismatch tolerance assays *in vitro* | pRSF-Duet1 derived plasmid containing type IV-A3 genes with C-terminally hexa-histidine tagged *csf2* (*cas7*) with a Δ177-224 (GSSG) mutation | Kanamycin |
| pRC043 | Mismatch tolerance assays *in vitro* | pACYCDuet-1 derived plasmid containing a protospacer mutation in positions 13-17 | Chloramphenicol |
| pRC044 | Mismatch tolerance assays *in vitro* | pACYCDuet-1 derived plasmid containing a protospacer mutation in positions 17-20 | Chloramphenicol |
| pRC045 | Mismatch tolerance assays *in vitro* | pACYCDuet-1 derived plasmid containing a protospacer mutation in positions 19-23 | Chloramphenicol |
| pRC046 | Mismatch tolerance assays *in vitro* | pACYCDuet-1 derived plasmid containing a protospacer mutation in positions 21-24 | Chloramphenicol |
| pRC047 | Mismatch tolerance assays *in vitro* | pACYCDuet-1 derived plasmid containing a protospacer mutation in position 25-28 | Chloramphenicol |
| pRC048 | Mismatch tolerance assays *in vitro* | pACYCDuet-1 derived plasmid containing a protospacer mutation in position 25-29 | Chloramphenicol |
| pRC049 | Mismatch tolerance assays *in vitro* | pACYCDuet-1 derived plasmid containing a protospacer mutation in position 31-32 | Chloramphenicol |
| pSJK35 | Phage targeting assays | pHERD30T derived plasmid containing repeat-spacer-repeat mini-CRISPR from type IV-A3 CRISPR-array with a spacer targeting *phage λ*-vir | Gentamicin |
| pSC179 | Phage targeting assays | pHERD30T derived plasmid containing containing a non-target sequence | Gentamicin |
| pSC031 | Phage targeting assays | pMMB-67-HE derived plasmid containing type IV-A3 genes | Ampicillin |
| pSC289 | Phage targeting assays | pMMB-67-HE derived plasmid containing type IV-A3 genes and encoding Cas7 with amino acid substitution W119A | Ampicillin |
| pSC290 | Phage targeting assays | pMMB-67-HE derived plasmid containing type IV-A3 genes and encoding Cas7 with amino acid substitution F95A | Ampicillin |
| pSC291 | Phage targeting assays | pMMB-67-HE derived plasmid containing type IV-A3 genes and encoding Cas8 with amino acid substitution K155A | Ampicillin |
| pSC292 | Phage targeting assays | pMMB-67-HE derived plasmid containing type IV-A3 genes and encoding Cas5 with amino acid substitution K119A | Ampicillin |
| pSC293 | Phage targeting assays | pMMB-67-HE derived plasmid containing type IV-A3 genes and encoding Cas5 with amino acid substitution R120A | Ampicillin |
| pSC294 | Phage targeting assays | pMMB-67-HE derived plasmid containing type IV-A3 genes and encoding Cas5 with amino acid substitution R121A | Ampicillin |
| pSC295 | Phage targeting assays | pMMB-67-HE derived plasmid containing type IV-A3 genes and encoding Cas8 with amino acid substitution V154A | Ampicillin |

| | | | |
|---|---|---|---|
| pSC296 | Phage targeting assays | pMMB-67-HE derived plasmid containing type IV-A3 genes and encoding Cas5 with amino acid substitution T114A | Ampicillin |
| pSC297 | Phage targeting assays | pMMB-67-HE derived plasmid containing type IV-A3 genes and encoding Cas5 with amino acid substitution L112A | Ampicillin |
| pSC298 | Phage targeting assays | pMMB-67-HE derived plasmid containing type IV-A3 genes and encoding Cas8 with amino acid substitution K79A | Ampicillin |
| pSC299 | Phage targeting assays | pMMB-67-HE derived plasmid containing type IV-A3 genes and encoding Cas8 with amino acid substitution Y51A | Ampicillin |
| pSC300 | Phage targeting assays | pMMB-67-HE derived plasmid containing type IV-A3 genes and encoding Cas5 with amino acid substitutions R102A, R104A, R105A, R110A | Ampicillin |

* plasmid sequences are provided as: Supplementary Data 2. Plasmid Sequences.

**Supplementary Tab. 5 |** Bacterial strains.

| **Strain** | **Genotype** | **Purpose** |
|---|---|---|
| *E. coli* Mach1 | *F- φ80(lacZ)ΔM15 ΔlacX74 hsdR(rK-mK+) ΔrecA1398 endA1 tonA* | Cloning |
| *E. coli* BL21 Star (DE3) | *F- ompT hsdSB (rB-mB-) gal dcm rne131 (DE3)* | Expression |
| *E. coli* DH5*α* | *F- endA1, glnV44, thi-1, recA1, relA1, gyrA96, deoR, nupG, purB20, φ80dlacZΔM15 Δ(lacZYA-argF), U169, hsdR17(rK–mK+), λ–* | Cloning |
| *E. coli* BL21(DE3) LOBSTR | *F– ompT gal dcm lon hsdSB*($r_B$–$m_B$–) *λ (DE3) [lacI lacUV5-T7p07 ind1 sam7 nin5]) [malB*$^+$*]K-12(λS)* | Expression |
| *E. coli* BL21-AI | *F– ompT gal dcm lon hsdSB(rB–mB–) [malB+]K-12(λS) araB::T7RNAP-tetA* | EOT, repression assays and expression |
| *P. oleovorans* DSM1045 | *wild type* | Reporter gene assays |
| *E. coli* WM3064 | *thrB1004 pro thi rpsL hsdS lacZΔM15 RP4-1360 Δ(araBAD)567 ΔdapA1341::[erm pir]* | Conjugation |
| *E. coli* GeneHogs | *F- mcrA Δ(mrr-hsdRMS-mcrBC) φ80lacZΔM15 ΔlacX74 recA1 araD139 Δ(araleu)7697 galU galK rpsL (StrR) endA1 nupG fhuA::IS2 (confers phage T1 resistance)* | Cloning, phage targeting assays |

## SUPPLEMENTARY FIGURES

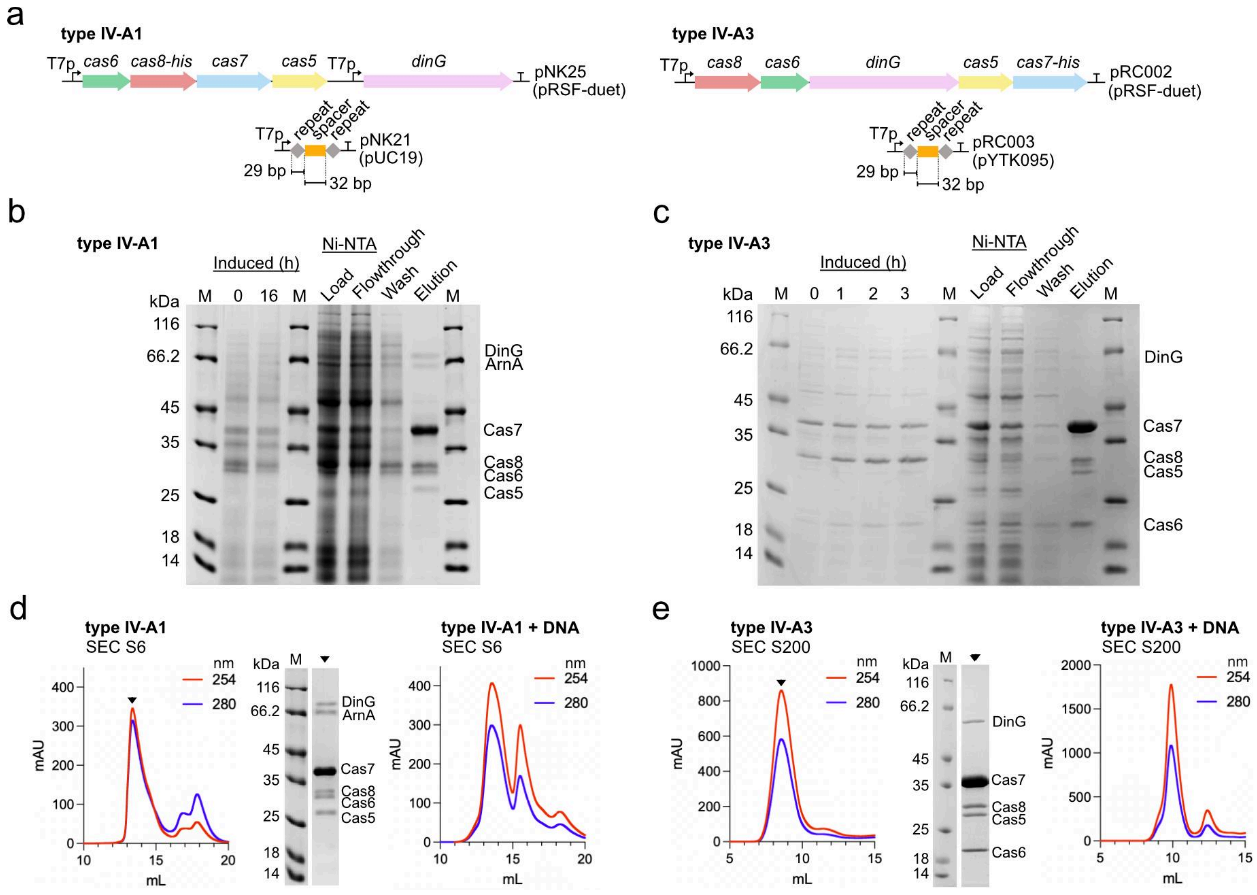


**Supplementary Fig. 1 | Reconstitution of DNA-bound type IV-A RNP complexes. a** Scheme illustrating type IV-A expression plasmids. **b** and **c,** SDS-PAGE of types IV-A1 (**b**) and IV-A3 (**c**) effector complex expression and Ni-NTA affinity chromatography samples. **d** SEC traces (S6 10/300) of binary (left) and DNA-bound ternary (right) type IV-A1 effector complexes. **e** SEC traces (S200 10/300) of binary (left) and DNA-bound ternary (right) type IV-A3 effector complexes.

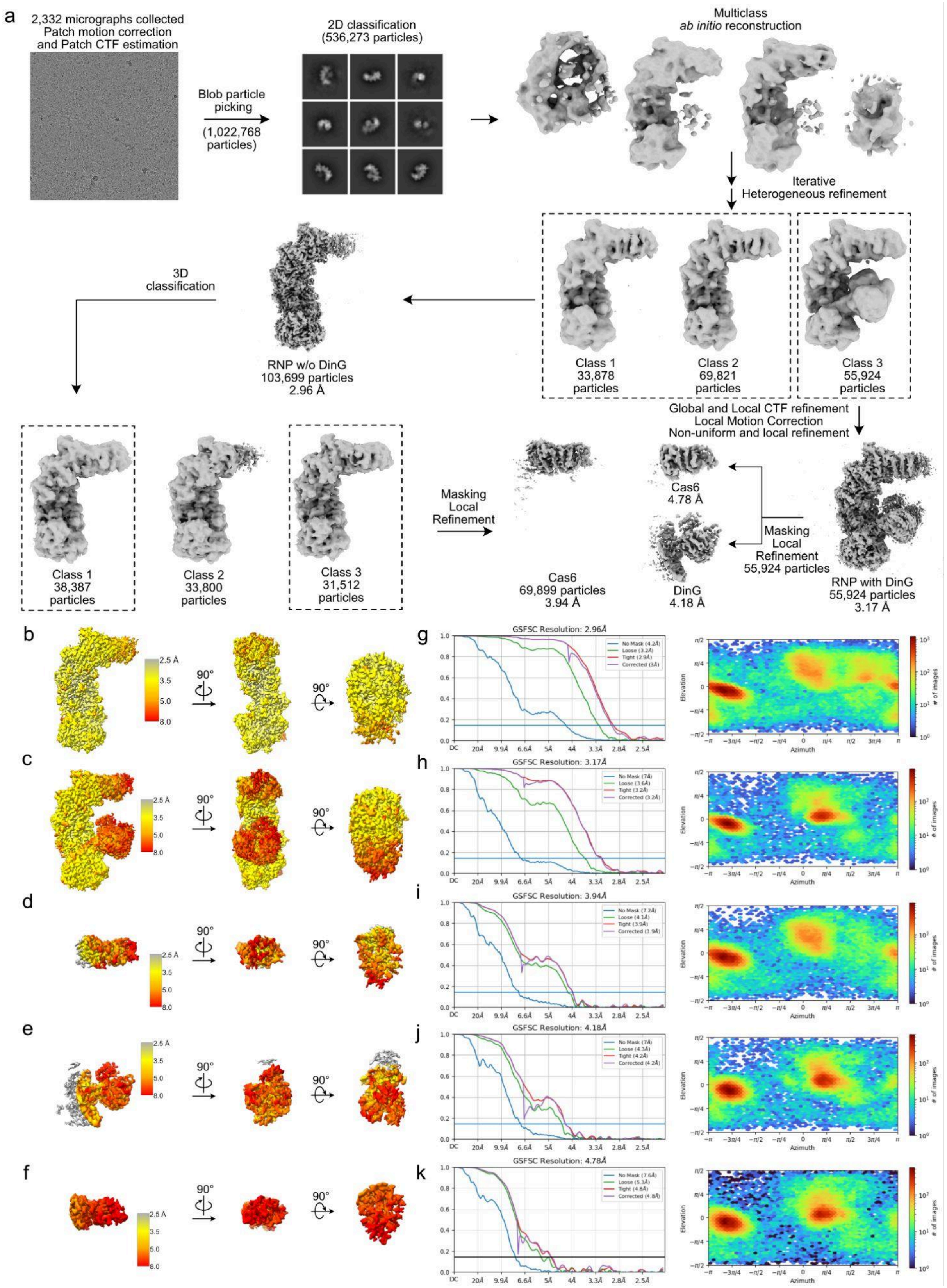


**Supplementary Fig. 2 | Cryo-EM data processing for type IV-A1 in complex with DNA. a** Processing workflow of the type IV-A1 dataset. All steps were performed in CryoSPARC (v. 4.2), except

for refinement of Cas6 in the DinG bound state (**f, k**), which was performed in CryoSPARC (v4.5). **b** and **c** Local resolution map, calculated in CryoSPARC (v. 4.2) for the type IV-A1 complex in the absence of DinG (**b**) and presence of DinG (**c**). **d-f** Local resolution focused maps of Cas6 (**d**), DinG (**f**) and Cas6 of the DinG bound complex (**f**). **g-k,** FSC curves and angular distribution plots calculated in CryoSPARC (v. 4.2 and 4.5) for type IV-A1 complex in absence of DinG (**g**), the focused map of Cas6 (**i**); the type IV-A1 complex with DinG bound (**h**), the focused maps of DinG (**j**), and Cas6 (**k**). The global resolution at FSC 0.143 is indicated by a solid blue line.

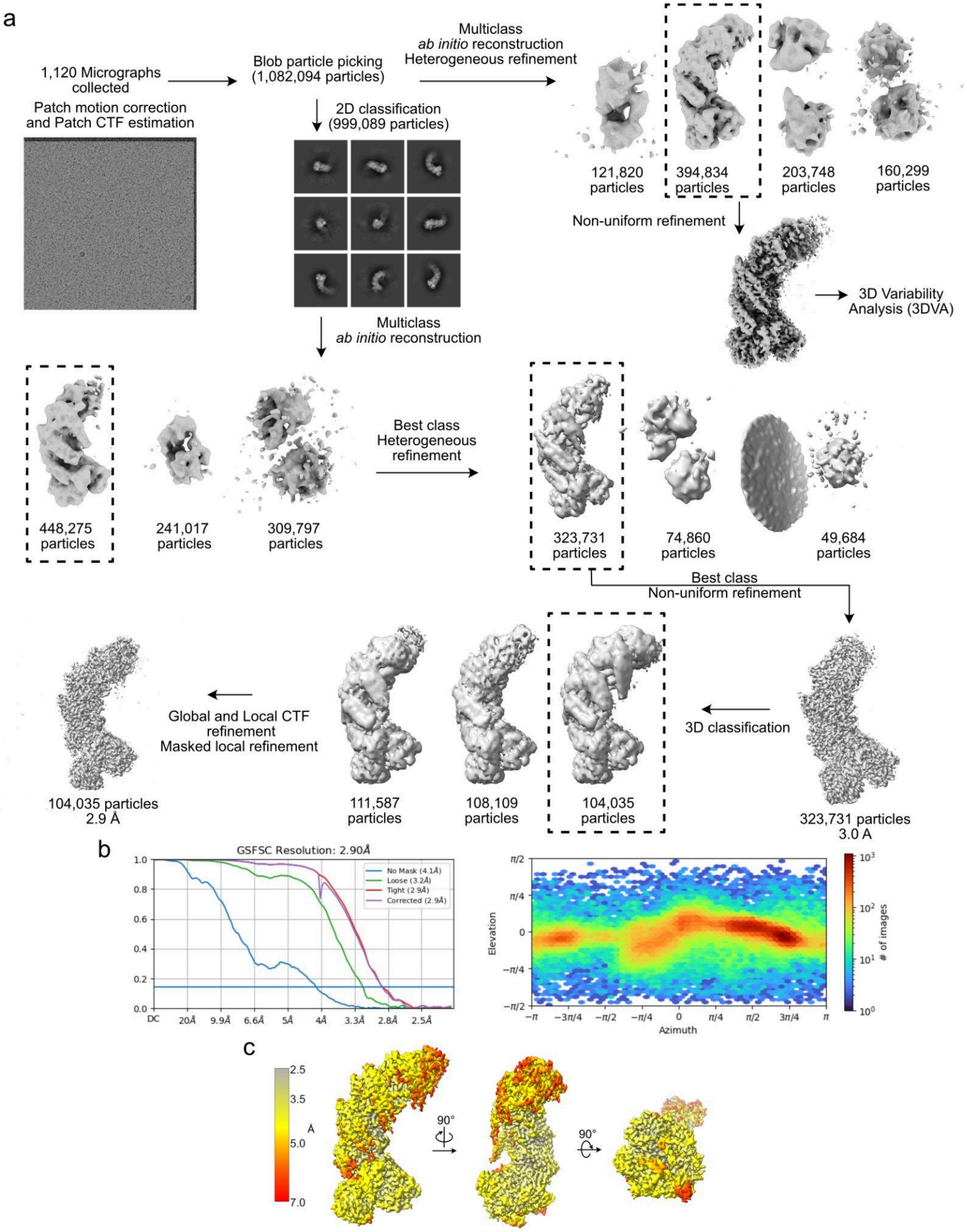


**Supplementary Fig. 3 | Cryo-EM data processing for type IV-A3 in complex with DNA. a** Processing workflow of the type IVA-3 DNA bound CRISPR complex. All steps performed in Cryosparc (v. 4.2). **b** FSC curve and angular distribution plot calculated in CryoSPARC (v. 4.2). The global resolution at FSC 0.143 is indicated by a solid blue line. **c** final reconstruction map colored according to local resolution.

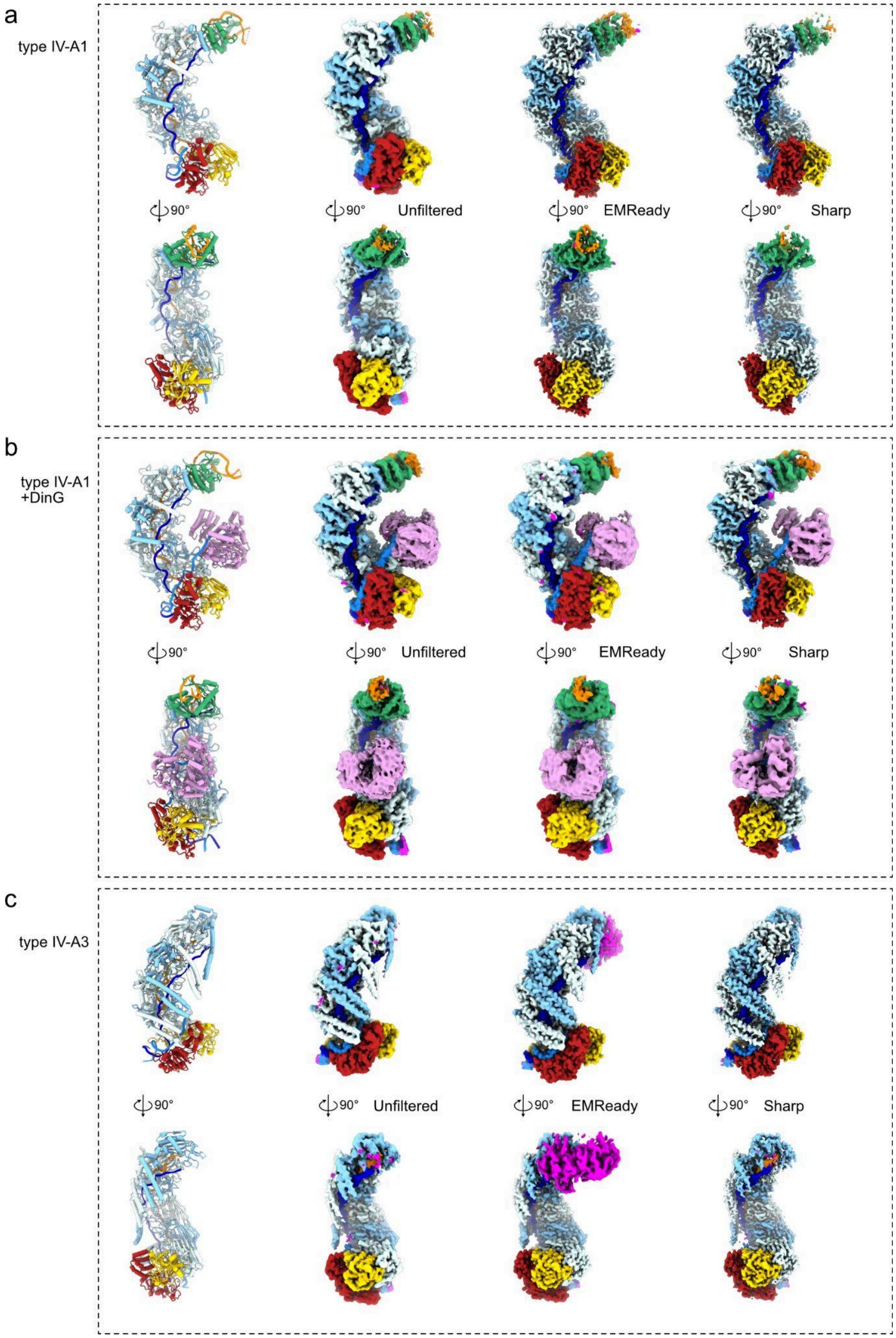


**Supplementary Fig. 4 | Coordinates, experimental and EMReady-processed maps of type IV-A1 with and without DinG, and type IV-A3 in absence of DinG.** Shown are model coordinates (cartoon) and maps (volumes) of type IV-A1 in absence (**a**) and presence of DinG (**b**), as well as type IV-A3 in absence of DinG (**c**). Experimental unfiltered and sharp maps, as well as EMReady processed maps are shown and labeled accordingly. The EMReady map in (**b**) is shown contoured at two different levels. Magenta volumes indicate unmodelled regions.

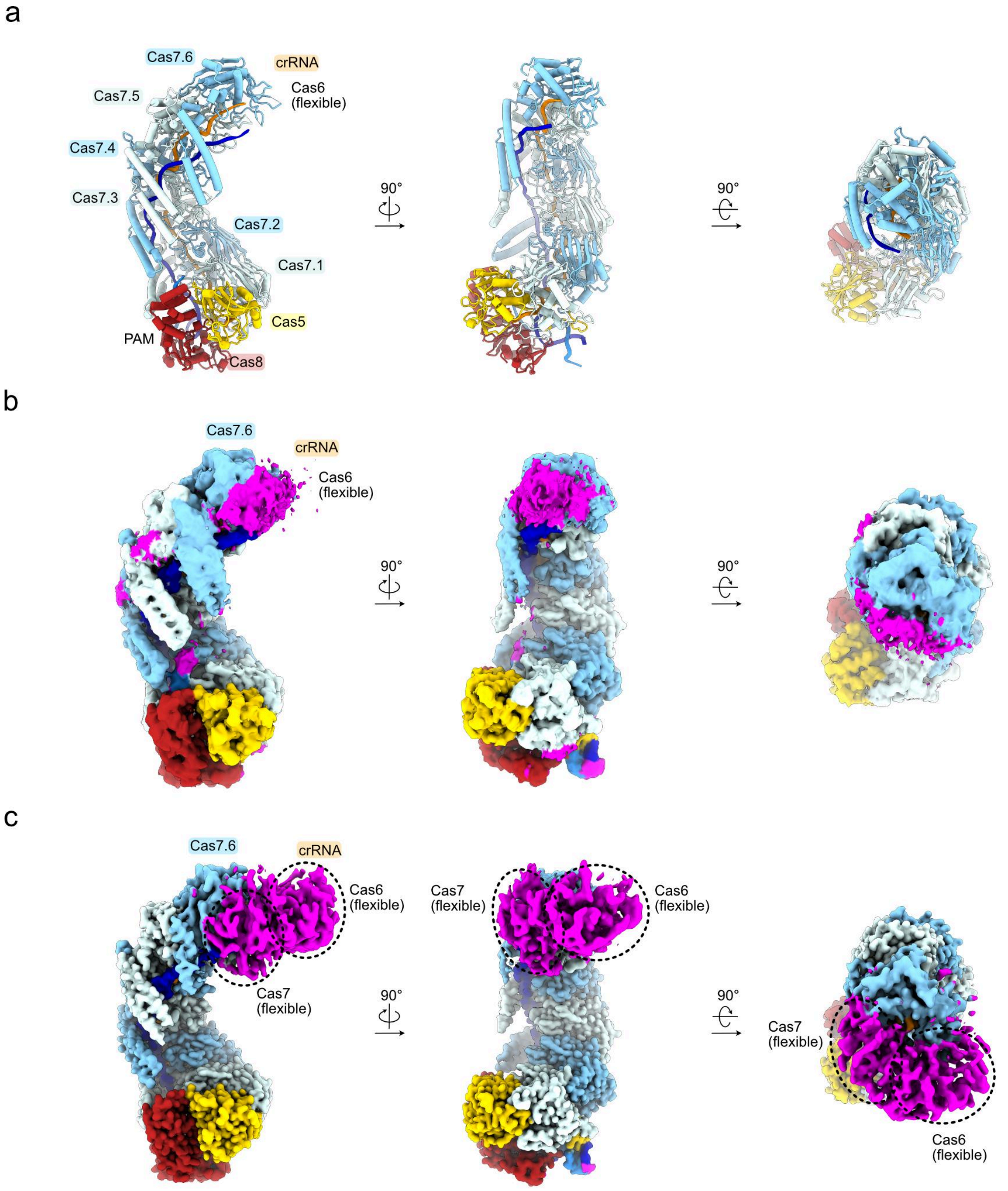


**Supplementary Fig. 5 | The terminal Cas7 and Cas6 are poorly resolved in type IV-A3. a** Structure model of the type IV-A3 effector bound to DNA in three 90°-rotated views. **b** and **c** Unfiltered (**b**) and EMReady (**c**) cryo-EM maps of the type IV-A3 effector in three 90°-rotated views, corresponding to the orientations shown in **a**. Magenta colored map segments highlight regions not covered by the model shown in **a**.

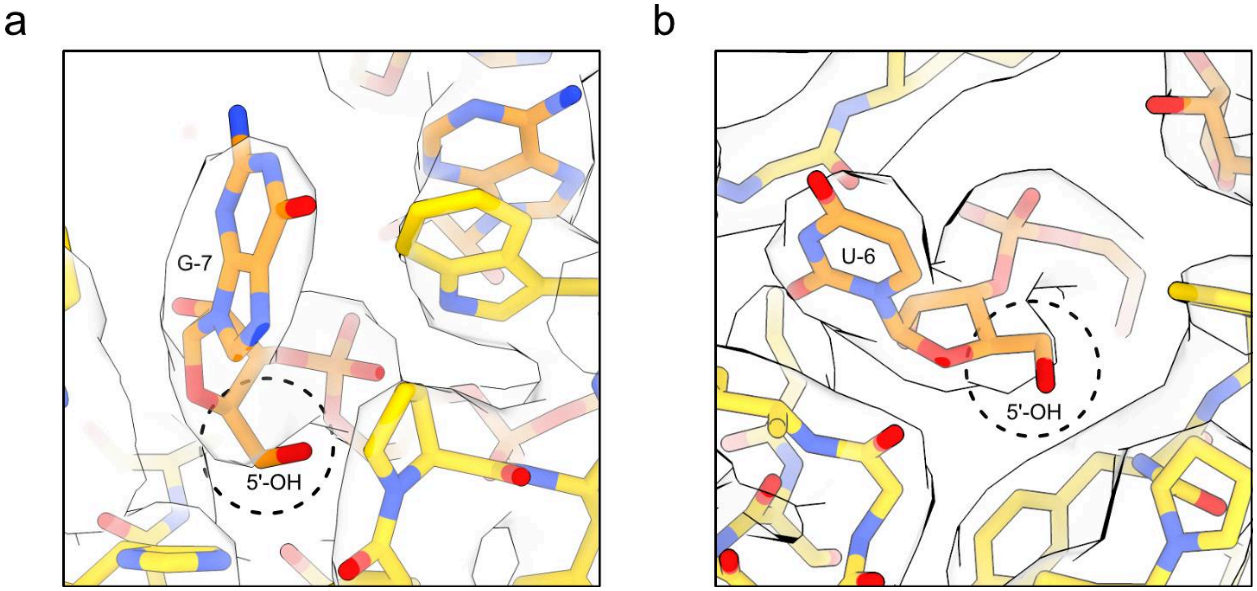


**Supplementary Fig. 6 | crRNA 5'-end chemistry in type IV-A1 and type IV-A3. a** Sharpened experimental cryo-EM map (white translucent surface) and model of the type IV-A1 effector showing the crRNA 5'-end (dashed circle). crRNA and Cas5 are shown as orange and yellow sticks, respectively. **b** Sharpened experimental cryo-EM map and model of the type IV-A3 effector showing the crRNA 5'-end (dashed circle). The crRNA and Cas5 are shown as orange and yellow sticks, respectively.

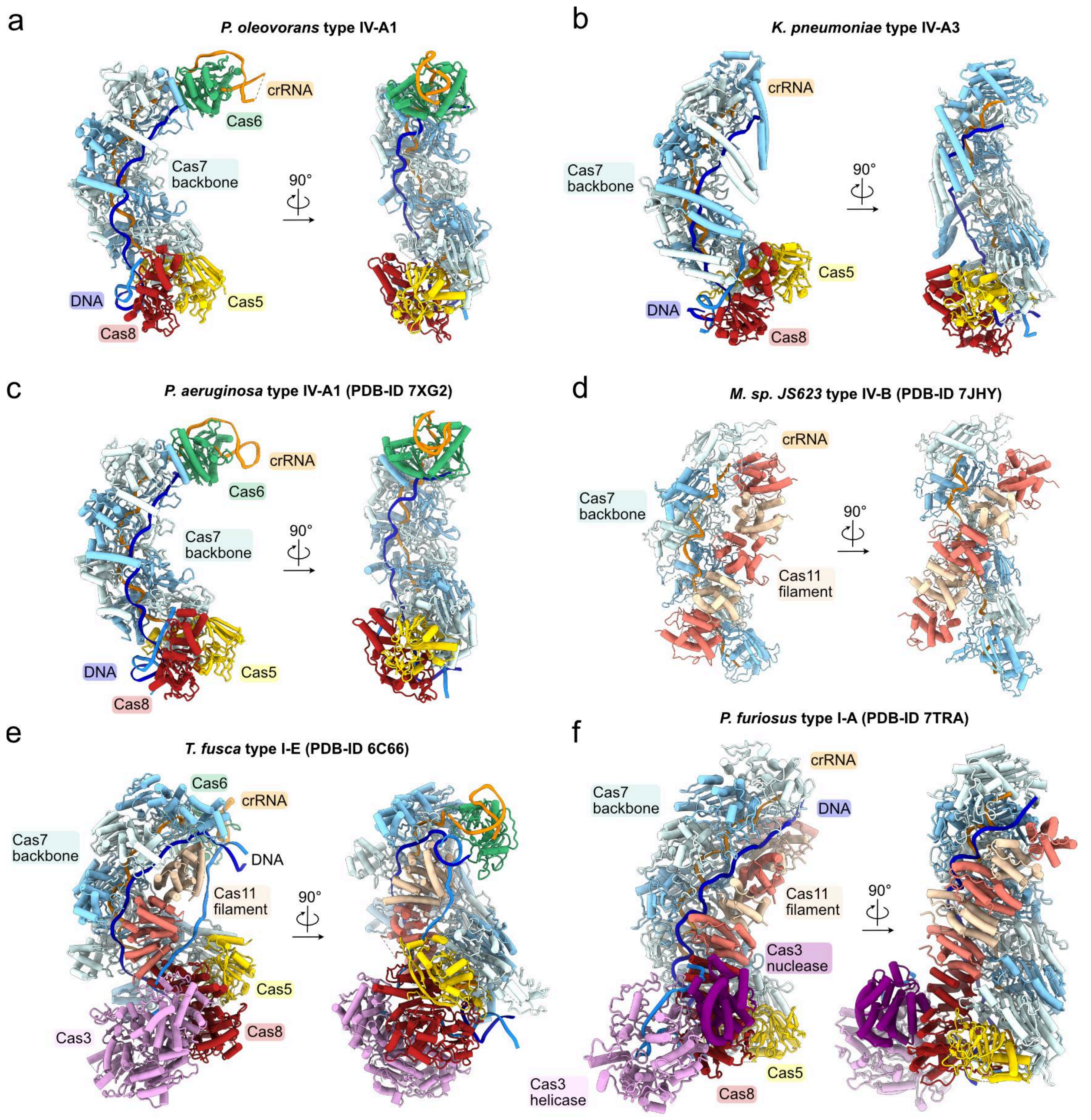


**Supplementary Fig. 7 | Comparison of type IV and type I effector structures. a** and **b** Type IV-A1 (**a**) and type IV-A3 (**b**) effector structures in complex with DNA (this study). **c** Cryo-EM structure of *P. aeruginosa* type IV-A1 in complex with DNA (PDB-ID 7XG2 [2]). **d** Partial cryo-EM structure of *M. sp. JS623* type IV-B in the absence of a cognate target (PDB-ID 7JHY [3]). **e** Cryo-EM structure of *T. fusca* type I-E in presence of DNA and Cas3 in the pre-nicking state (PDB-ID 6H66 [4]). **f** Cryo-EM structure of *P. furiosus* type I-A effector-Cas3 in presence of DNA (PDB-ID 7TRA [5]).

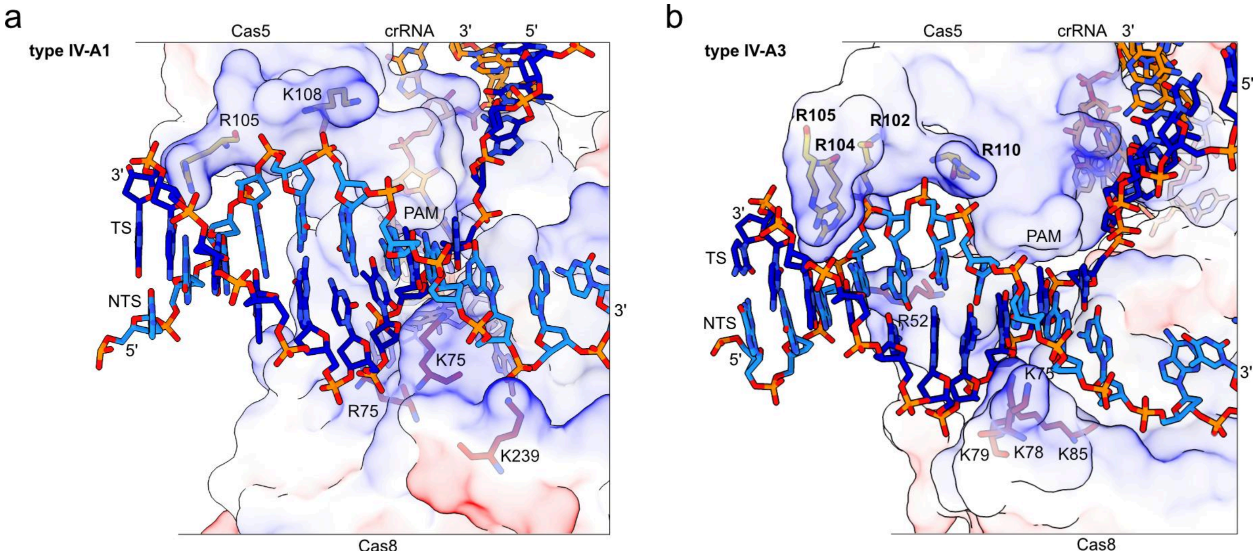


**Supplementary Fig. 8 | Cas8 and Cas5 form a positively charged vice to recruit the PAM. a** and **b** Shown is the PAM proximal dsDNA segment bound to the positively charged vice in the type IV-A1 effector (**a**) and the type IV-A3 effector (**b**). Blue surfaces indicate positive, and red surfaces indicate negative polarization. Arginine and lysine residues of Cas5 (yellow) and Cas8 (red) contacting the dsDNA segment are shown as yellow and red sticks, respectively. Amino acids producing interference defects upon substitution are highlighted in bold (data is shown in Fig. 2e).

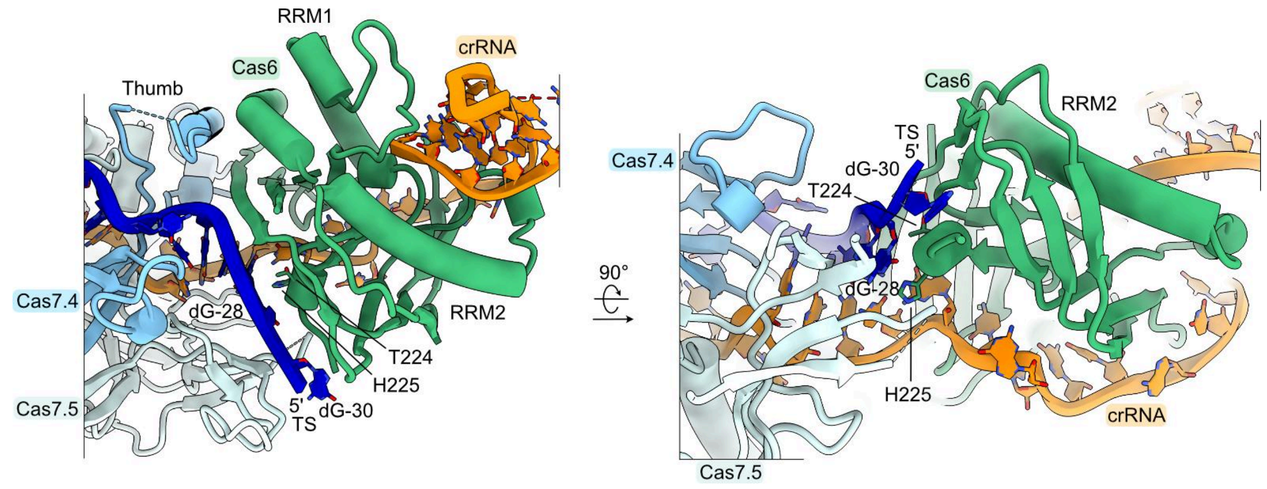


**Supplementary Fig. 9 | Type IV-A1 Cas6 caps the crRNA:TS hybrid.** Shown is a Cas6-centered view on the head of the type IV-A1 effector bound to DNA in two 90°-rotated orientations. Cas6 side chains in proximity to the terminal crRNA:TS base pair in position 28 are shown as sticks and labeled according to their identity.

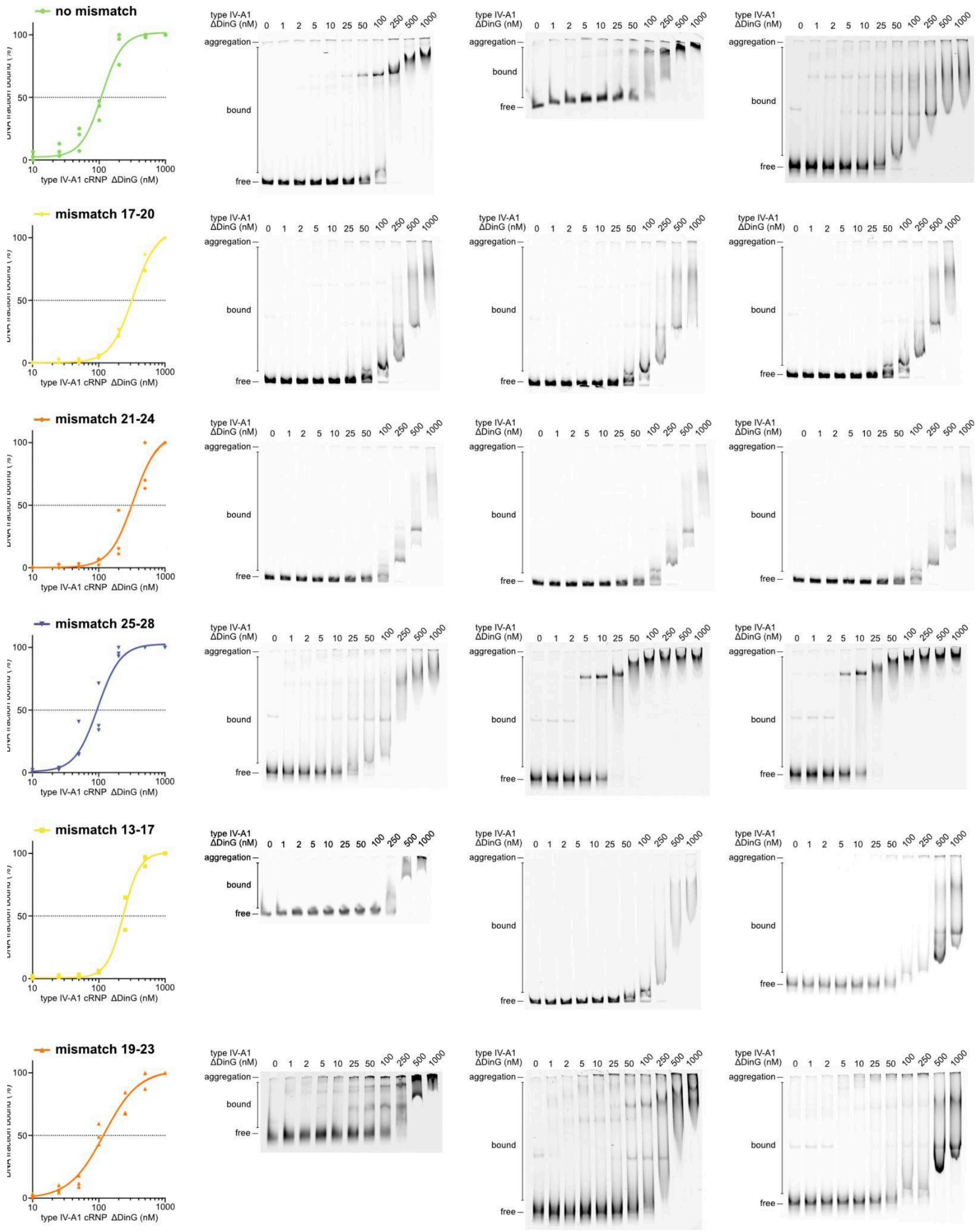


**Supplementary Fig. 10 | EMSA assay probing the ability of type IV-A1 to bind mismatched DNA.** Left: Graph showing the DNA binding efficiencies. Coloring corresponds to main text Figure 4E. Right: EMSA gels.

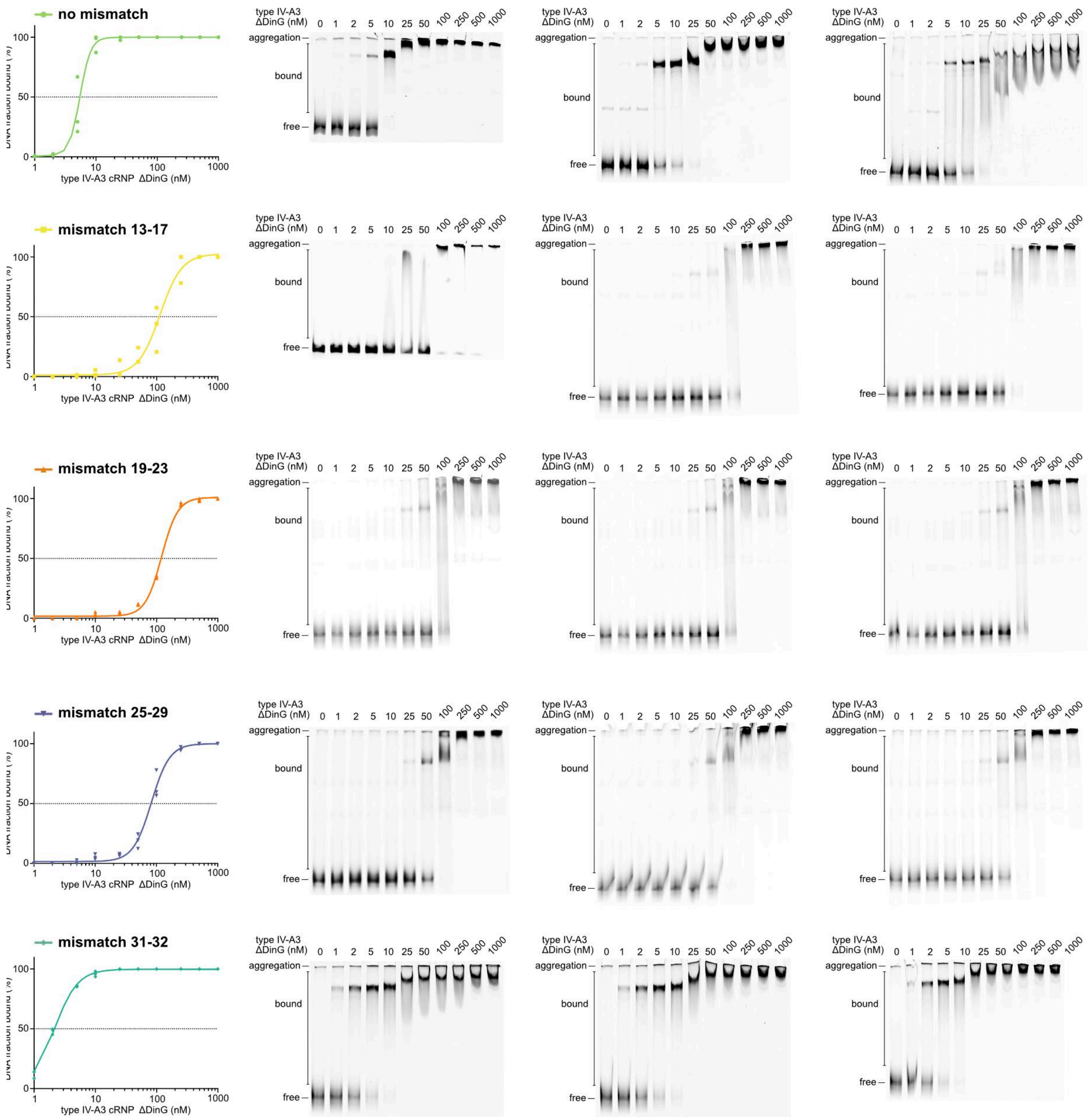


**Supplementary Fig. 11 | EMSA assay probing the ability of type IV-A3 to bind mismatched DNA.** Left: Graph showing the DNA binding efficiencies. Coloring corresponds to main text Figure 4F. Right: EMSA gels.

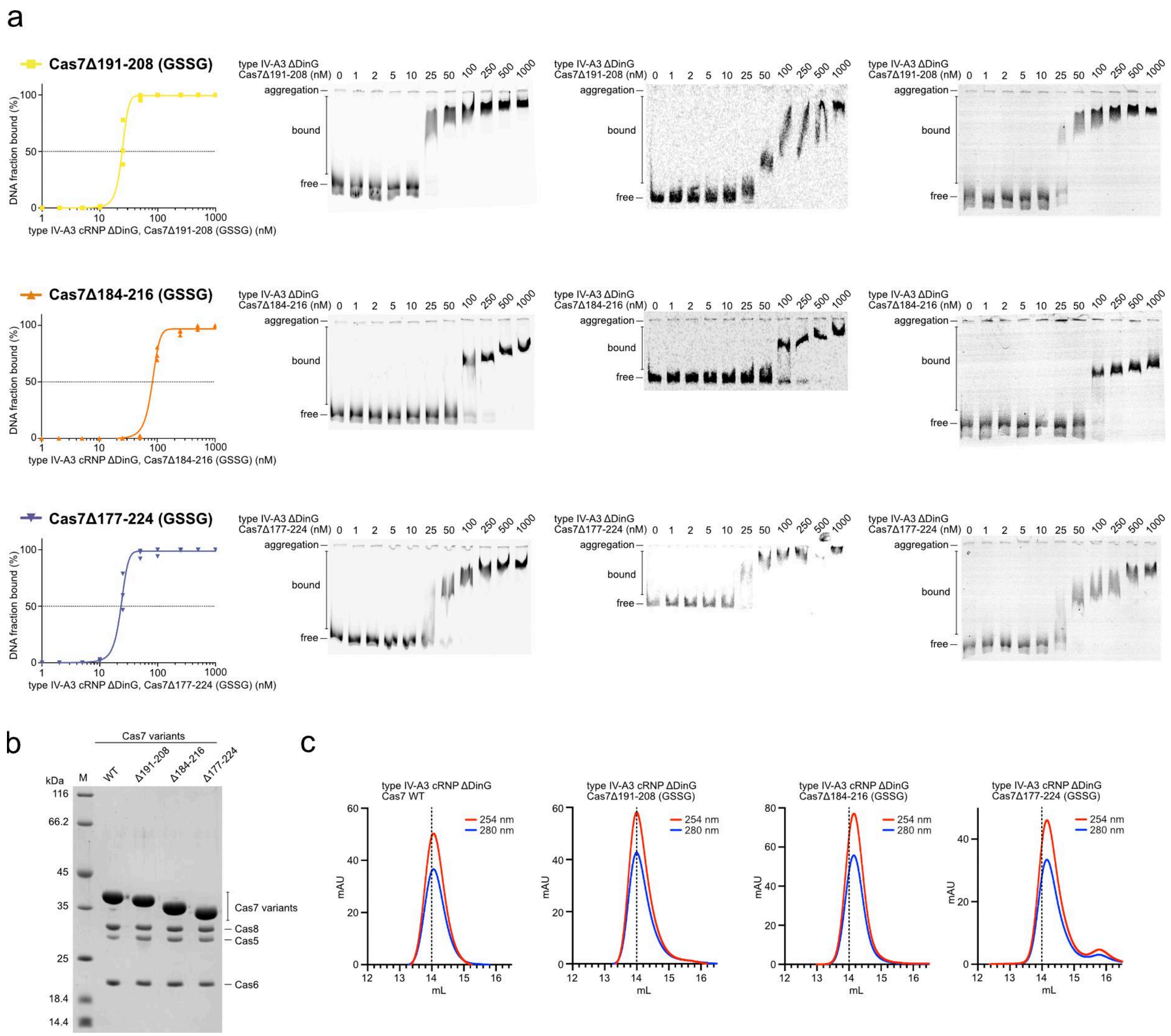


**Supplementary Fig. 12 | EMSA assay probing the ability of type IV-A3 complexes containing Cas7 variants to bind DNA. a** Left: Graph showing the DNA binding efficiencies. Coloring corresponds to main text Fig. 5d. Right: EMSA gels. **b** SDS-PAGE assessing the homogeneity of purified complexes. **c** Analytical size exclusion chromatography of type IV-A3 complexes containing Cas7 variants.

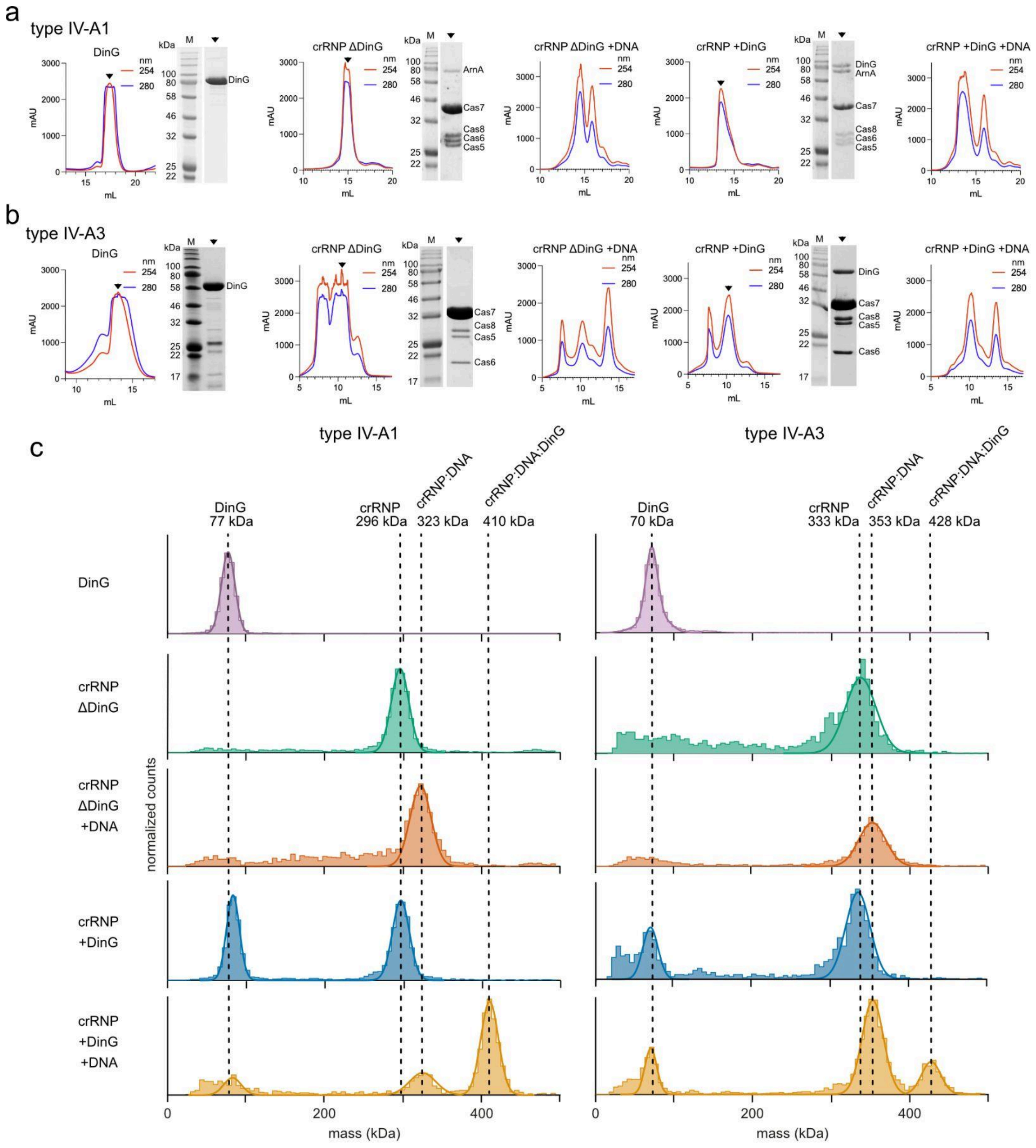


d

| Type | IV-A1 | | | | IV-A3 | | | |
|---|---|---|---|---|---|---|---|---|
| Analyte | DinG | crRNP | crRNP: DNA | crRNP: DNA:DinG | DinG | crRNP | crRNP: DNA | crRNP: DNA:DinG |
| Theoretical MW (kDa) | 77.88 | 283.96 | 321.63 | 399.51 | 69.92 | 327.18 | 364.24 | 434.16 |
| Measured MW (kDa) | ~77 | ~296 | ~323 | ~410 | ~70 | ~333 | ~353 | ~428 |

**Supplementary Fig. 13 | Mass photometry of purified and reconstituted type VI-A complexes. a** and **b** Preparative SEC traces of DinG and type IV CRISPR nucleoprotein (crRNP) complexes in the absence and presence of DinG and DNA of types IV-A1 (**a**) and IV-A3 (**b**), run on S6 10/300 and S200 10/300

columns, respectively. Triangles indicate fractions analyzed via SDS-PAGE. **c** Mass photometric analysis of peak fraction from **a** and **b**. Dashed lines highlight the measured molecular weight of representative data. Measurements were repeated three times with similar results. **d** Table listing the theoretical and measured molecular weights of the analytes.

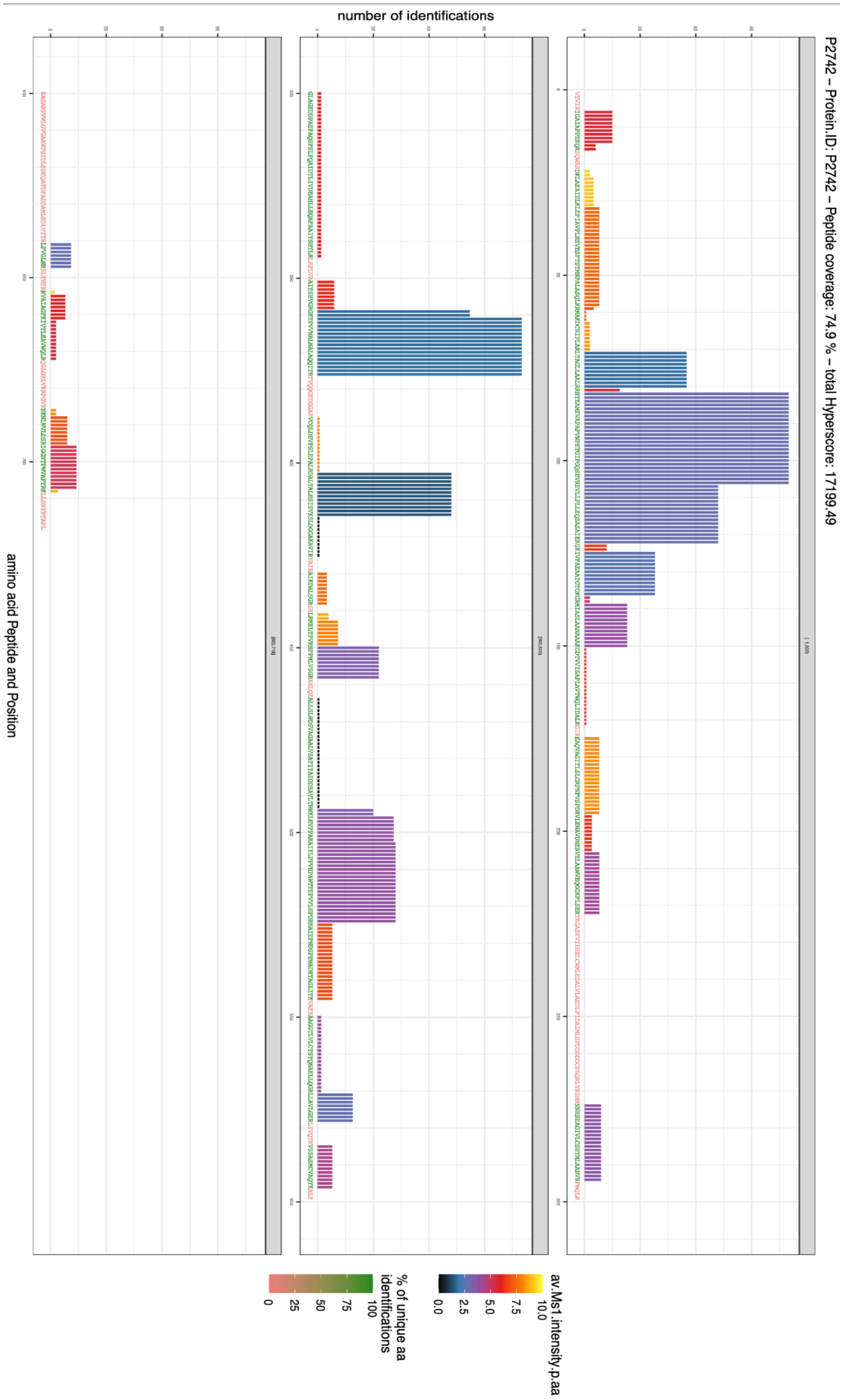


**Supplementary Fig. 14 | Mass spectrometry analysis of type IV-A1 DinG.** Shown are the number of peptide identification, intensities and % unique identifications for DinG (protein ID P2742). *n* = 1.

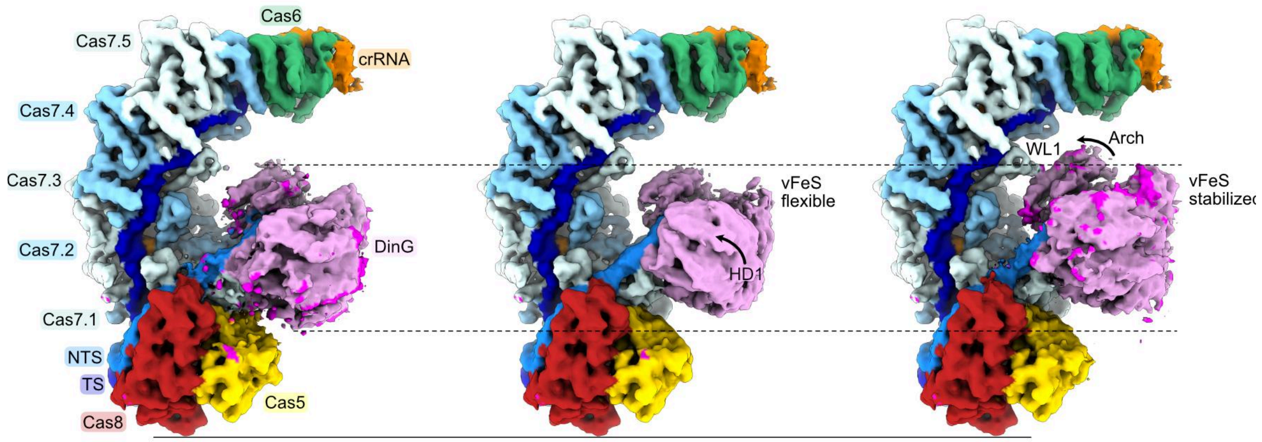


**Supplementary Fig. 15 | 3D variability analysis (3DVA) of type IV-A1 effector bound to DNA and DinG.** Shown are three representative frames of the 3DVA. Left: frame 1; center: frame 5; right: frame 10. Lines are shown to highlight the relative rearrangements in DinG. Arrows indicate domain rearrangements. Magenta colored map regions indicate densities not accounted for by our model.

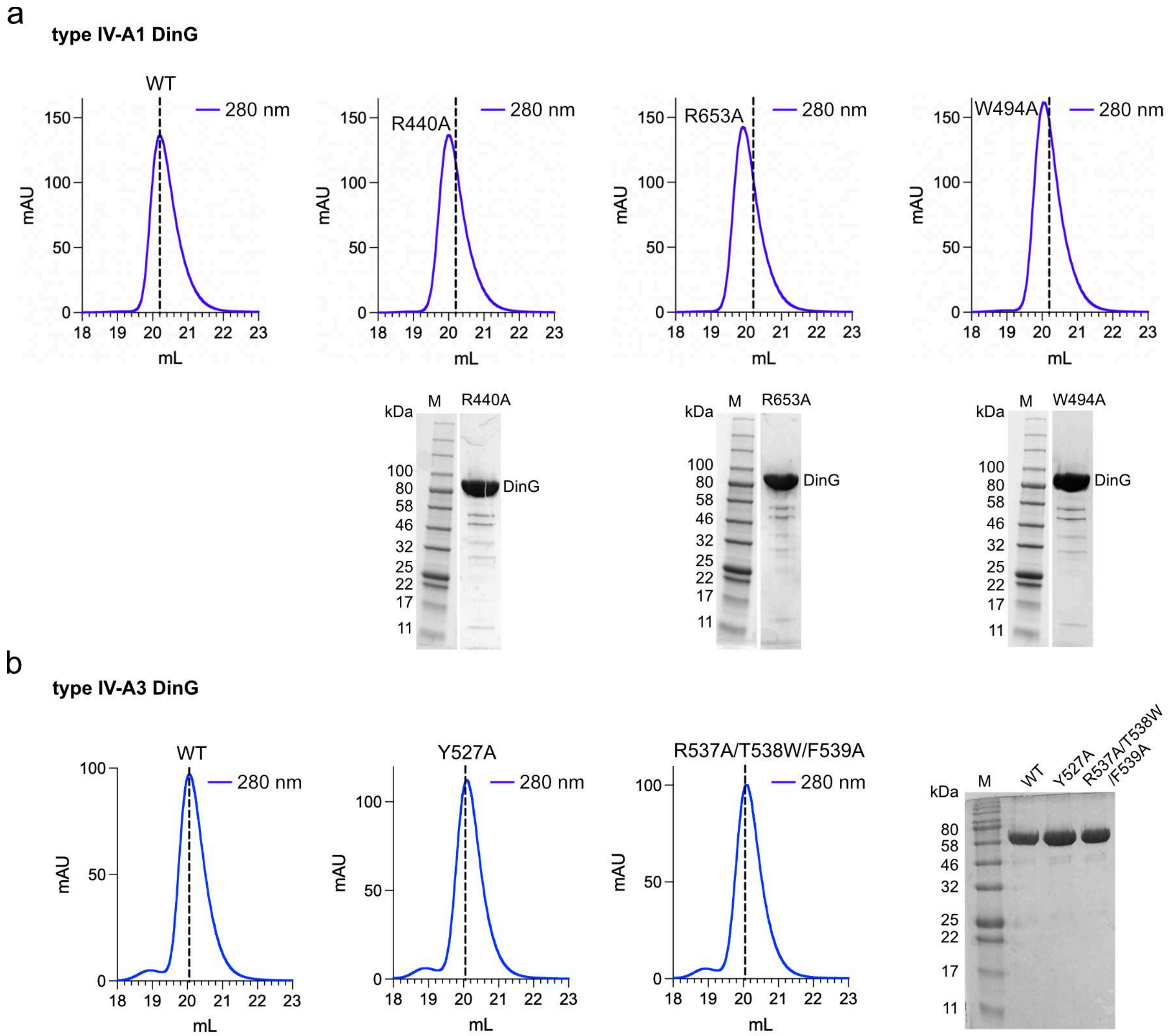


**Supplementary Fig. 16 | Variant DinG proteins elute as single peaks during analytical size-exclusion chromatography. a** Above: SEC traces of type IV-A1 variant DinG in comparison to WT DinG (left), recorded at 280 nm. Below: SDS-PAGEs of preparative SEC fractions, used for analytical SEC runs. An SDS-PAGE for WT is shown in supplementary fig. 10A. **b** Center: SEC traces of type IV-A3 variant DinG in comparison to WT DinG (left). Right: SDS-PAGEs of preparative SEC fractions, used for analytical SEC runs.

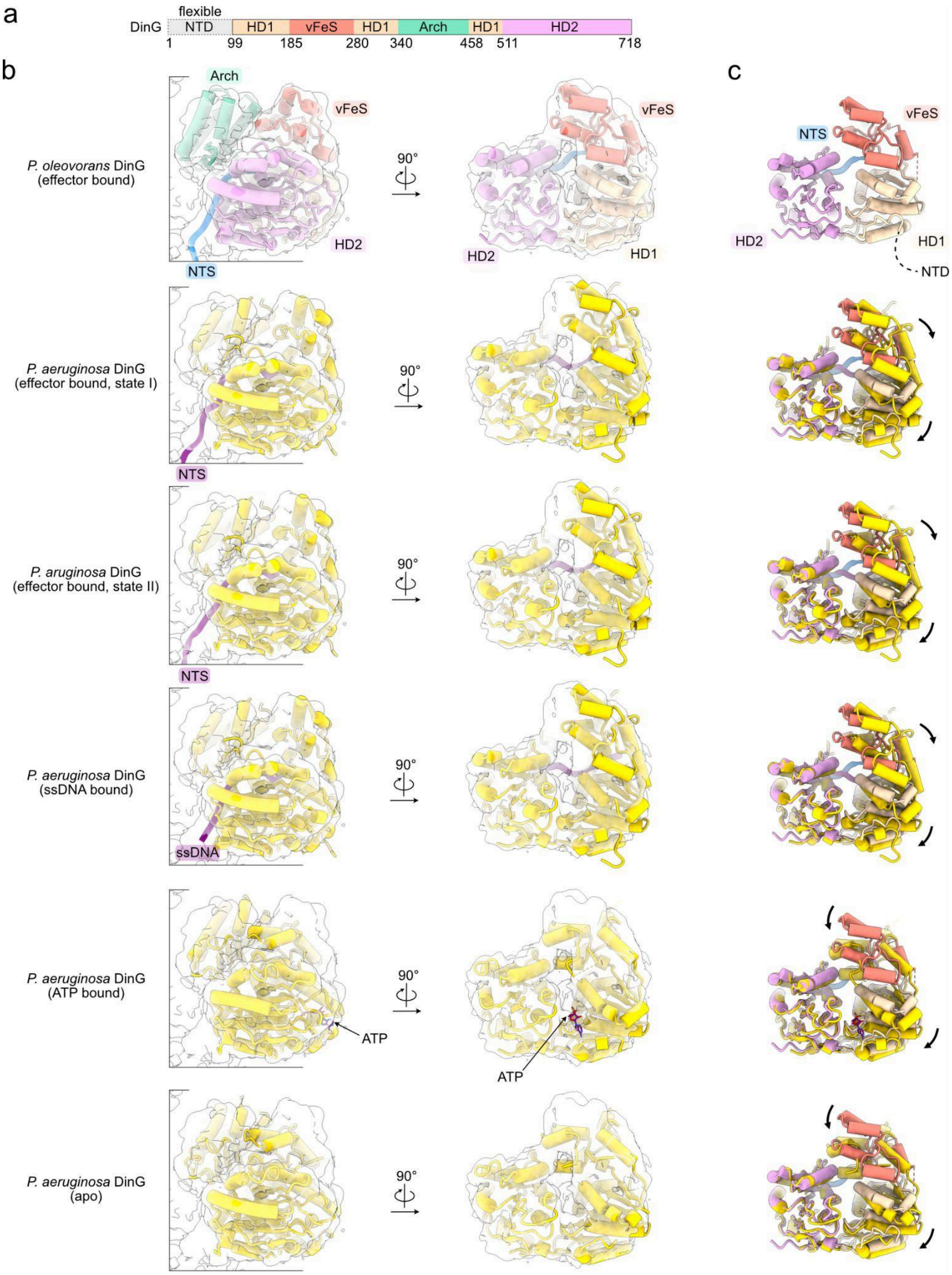


**Supplementary Fig. 17 | Comparison of type IV-A1 DinG states. a** Domain organization scheme of DinG. **b** Sharpened experimental cryo-EM map of the *P. oleovorans* type IV-A1 effector bound to DinG (

translucent white surface) and DinG states observed in this study (top, domain coloring as in **a**); and in supplementary ref. 2: PDB-IDs: 7XEX-A (apo), 7XF0-A (ATP), 7XF1 (ssDNA), 7XG3 (effector, state I), 7XG4 (effector state II), with DinG colored in yellow and nucleic acids in purple. Structures were superimposed according to the HD2 domain to highlight relative rearrangements. Arrows indicate domain rearrangements observed between the superimposed states.

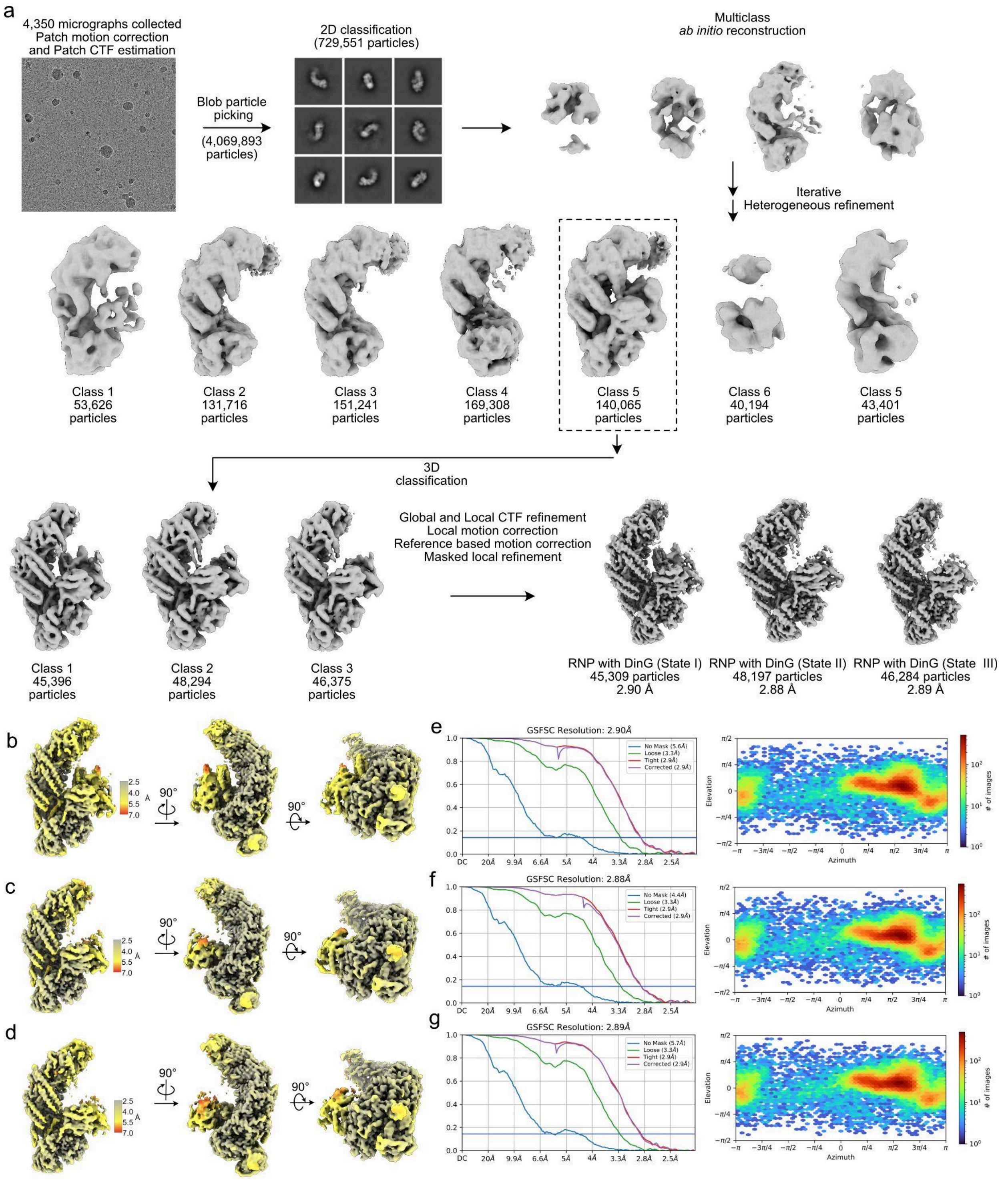


**Supplementary Fig. 18 | Cryo-EM data processing for type IV-A3 in complex with DinG. a** Processing workflow of the DNA-bound type IV-A3 effector complex in presence of DinG. All steps performed in CryoSPARC (v. 4.4). **b-d** Local resolution map, calculated in CryoSPARC (v. 4.4) for the type IV-A3 complex in the presence of DinG state I (**b**), state II (**c**), state III (**d**). **e-g** FSC curves calculated in CryoSPARC (v. 4.4) and orientation distribution of the final set of refined particles for state I (**e**), state II (**f**), state III (**g**). The global resolution at 0.143 is indicated by a solid blue line.

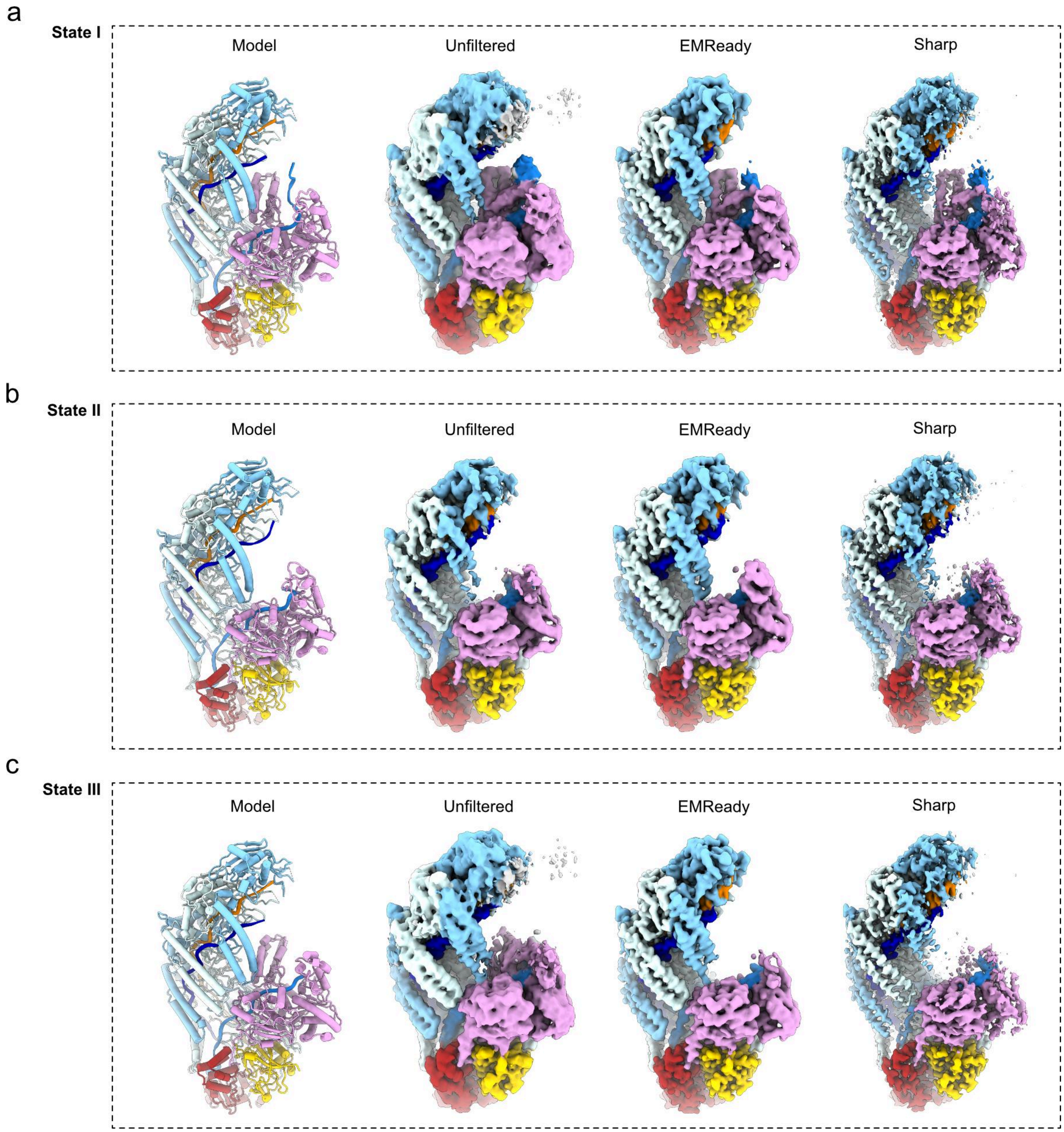


**Supplementary Fig. 19 | Coordinates, experimental and EMReady-processed maps of type IV-A3 in presence of DinG. a**, **b** and **c** Shown are model coordinates (cartoon) and maps (volumes) of type IV-A3 in presence of DinG for states I (**a**), II (**b**) and III (**c**). Experimental unfiltered and sharp maps, as well as machine learning processed maps (EMReady) are shown and labeled accordingly.

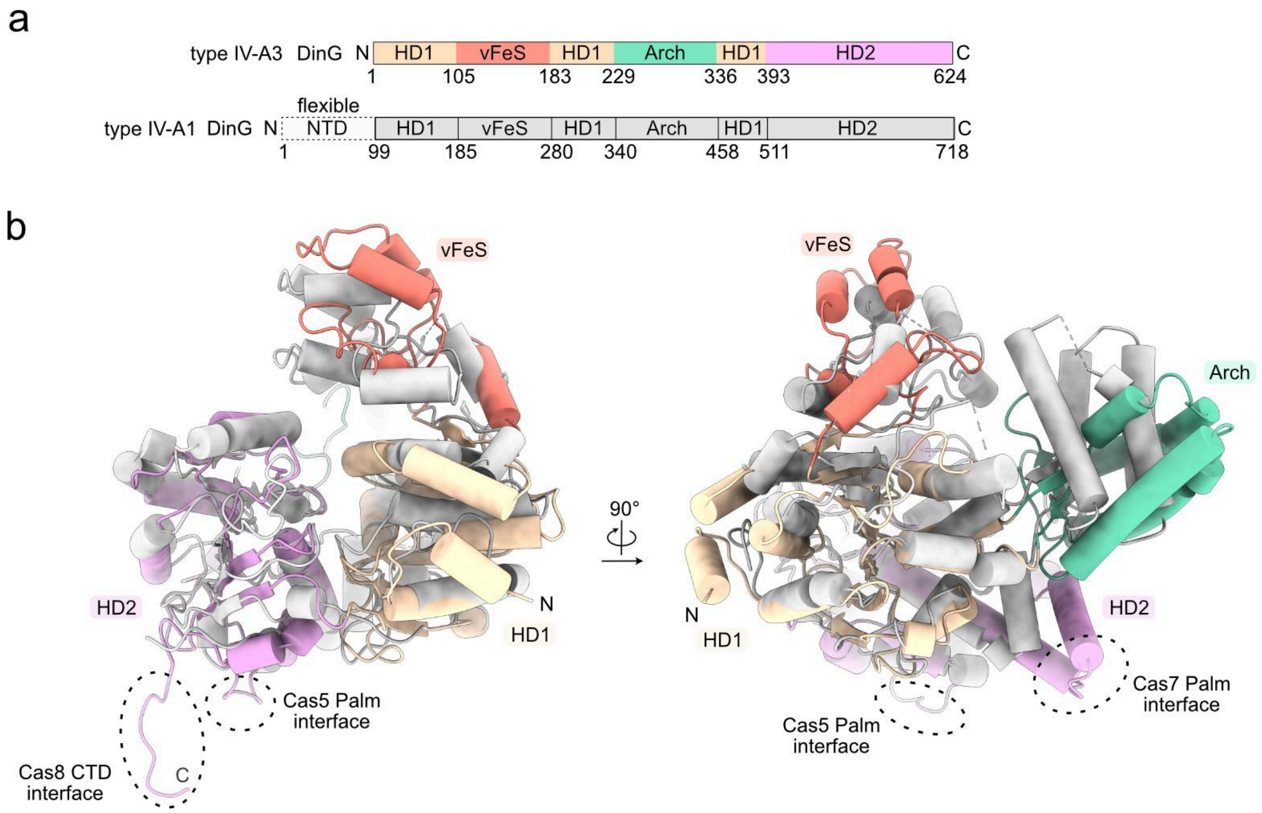


**Supplementary Fig. 20 | Comparison of type IV-A1 and type IV-A3 DinG. a** Domain organization scheme of DinG. **b** Superimposition of types IV-A1 and IV-A3 DinG (state I), as observed in complexes with DNA and the crRNP (not shown for clarity). Coloring as in **a**. crRNP interaction interfaces of type IV-A3 are indicated by dashed circles.

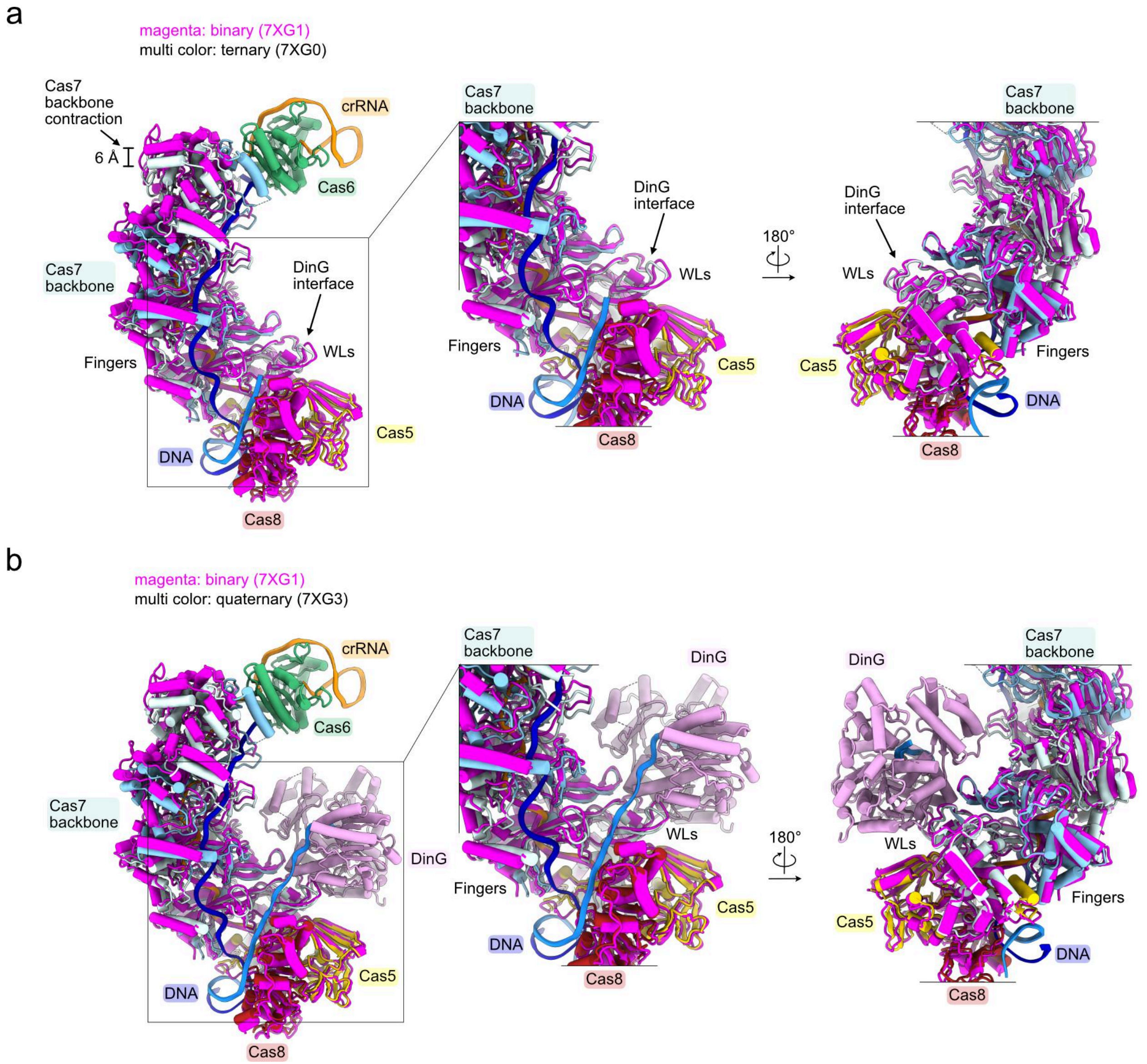


**Supplementary Fig. 21 | Superimposition of *P. aeruginosa* type IV-A1 structures. a** Left: superimposition of the binary (magenta, PDB-ID 7XG1 [2]) and ternary states (colored according to subunits, PDB-ID 7XG0 [2]). Structures were aligned based on Cas7.1. Right: Close-up view on the DinG interface in two 180°-rotated orientations. **b** Left: Superimposition of the binary (magenta, PDB-ID 7XG1 [2]) and quaternary states (colored according to subunits, PDB-ID 7XG3 [2]). Structures were aligned based on Cas7.1. Right: close-up view on the DinG interface in two 180°-rotated orientations.

# SUPPLEMENTARY REFERENCES


1. Benz, F. *et al.* Type IV-A3 CRISPR-Cas systems drive inter-plasmid conflicts by acquiring spacers in trans. *bioRxiv* 2023.06.23.546257 (2023) doi:10.1101/2023.06.23.546257.
2. Cui, N. *et al.* Type IV-A CRISPR-Csf complex: Assembly, dsDNA targeting, and CasDinG recruitment. *Mol. Cell* **83**, 2493–2508.e5 (2023).
3. Zhou, Y. *et al.* Structure of a type IV CRISPR-Cas ribonucleoprotein complex. *iScience* **24**, 102201 (2021).
4. Xiao, Y., Luo, M., Dolan, A. E., Liao, M. & Ke, A. Structure basis for RNA-guided DNA degradation by Cascade and Cas3. *Science* **361**, (2018).
5. Hu, C. *et al.* Allosteric control of type I-A CRISPR-Cas3 complexes and establishment as effective nucleic acid detection and human genome editing tools. *Mol. Cell* **82**, 2754–2768.e5 (2022).

## SUPPLEMENTARY SOURCE DATA

### Supplementary Fig. 1b

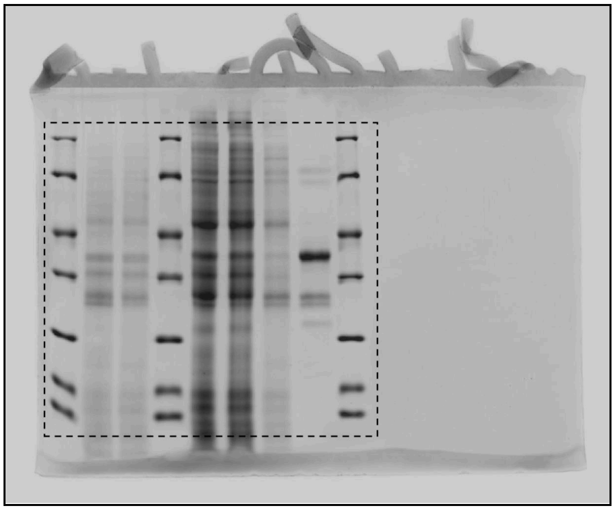

### Supplementary Fig. 1c

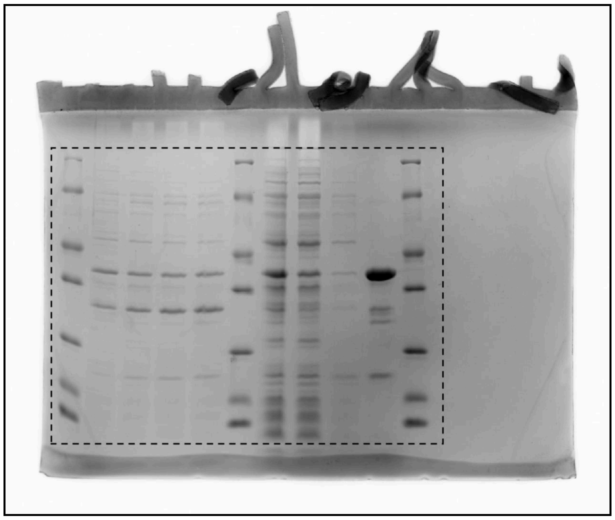

**Supplementary Fig. 1d**

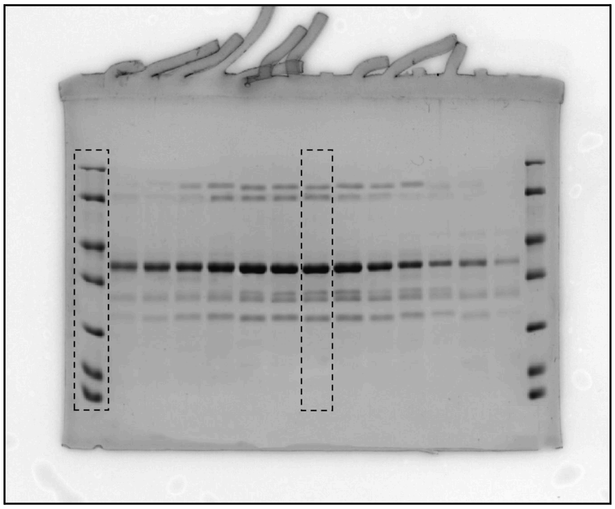

**Supplementary Fig. 1e**

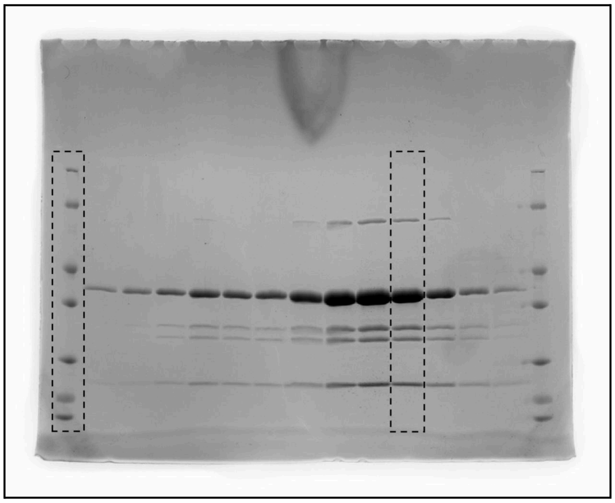

**Supplementary Fig. 10**

no mismatch; left panel

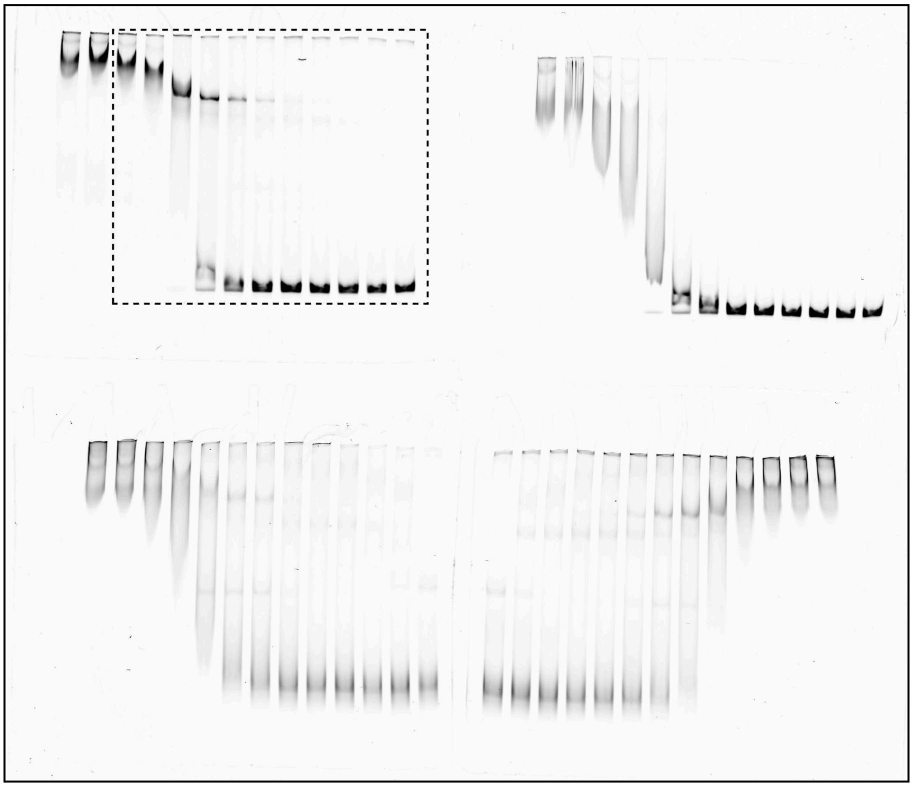

no mismatch; center panel

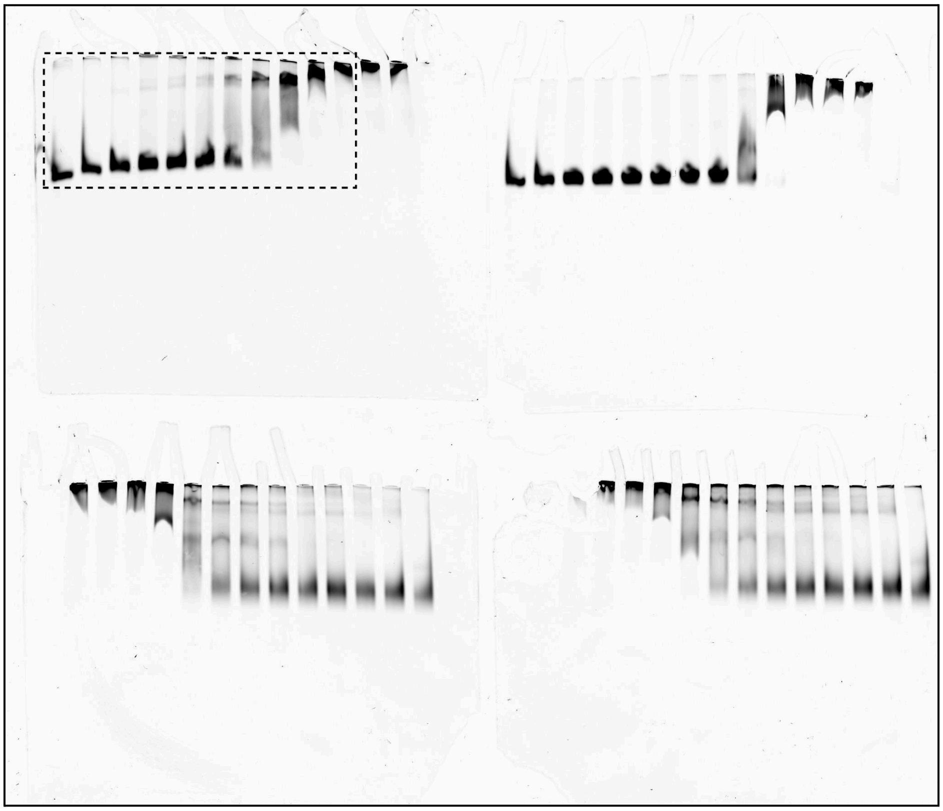

no mismatch; right panel

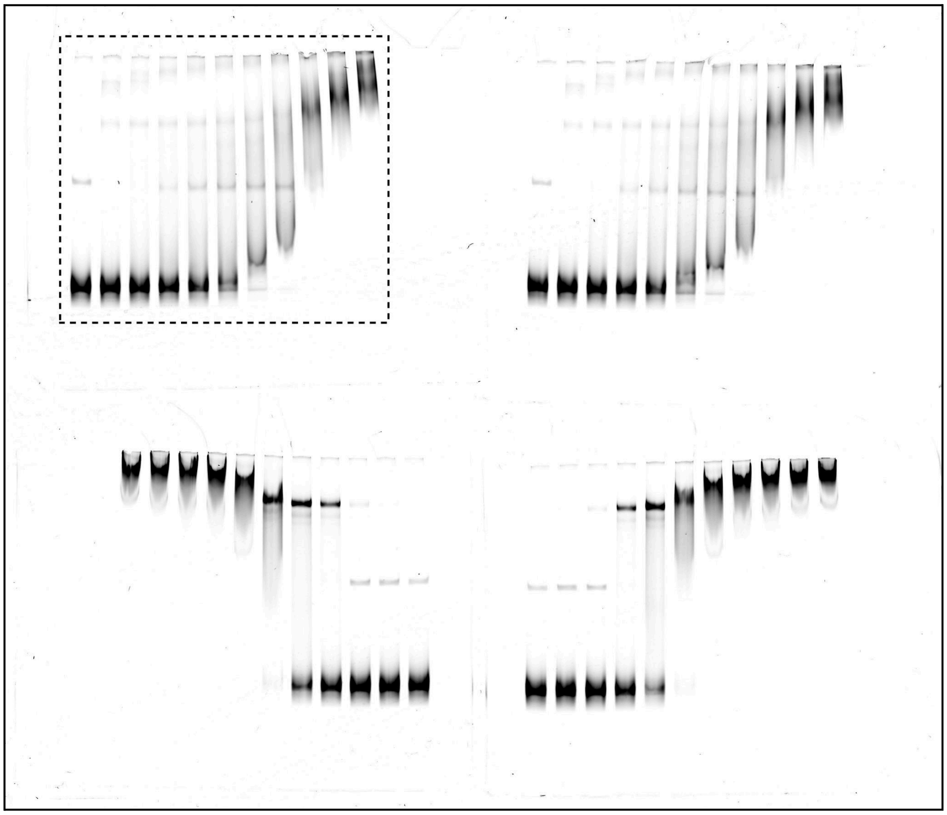

mismatch 17-20; left, center and right panel

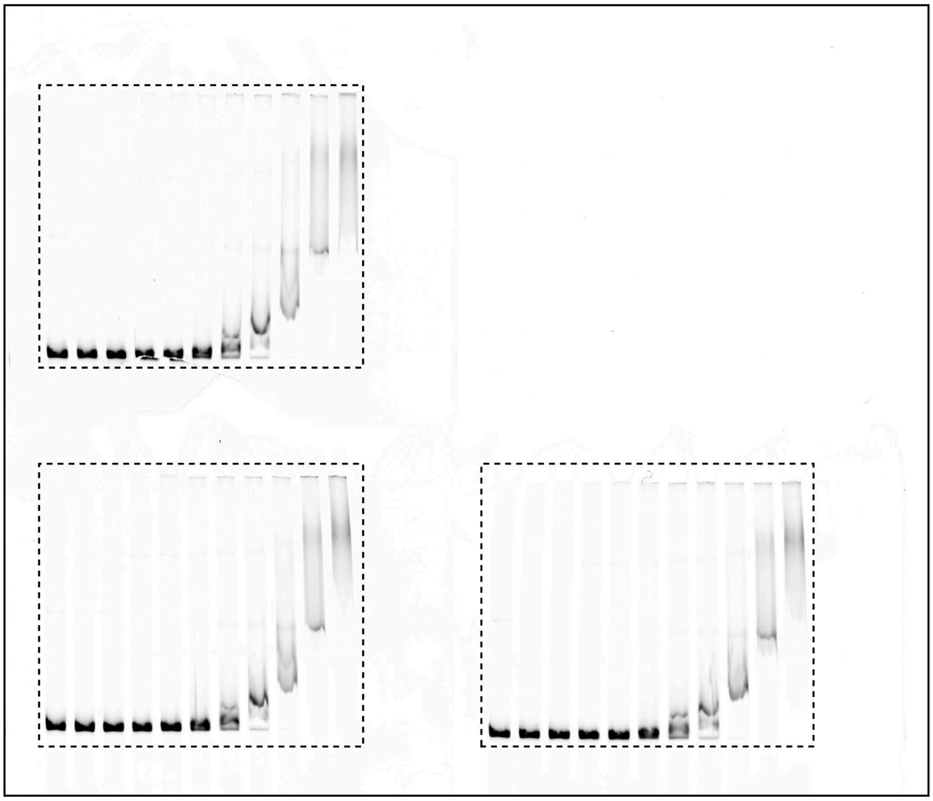

mismatch 21-24; left, center and right panel

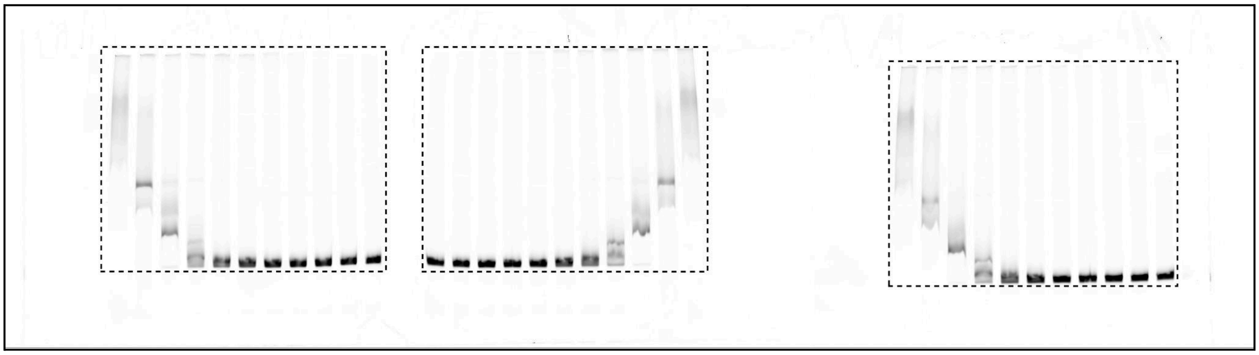

mismatch 25-28; left, center and right panel

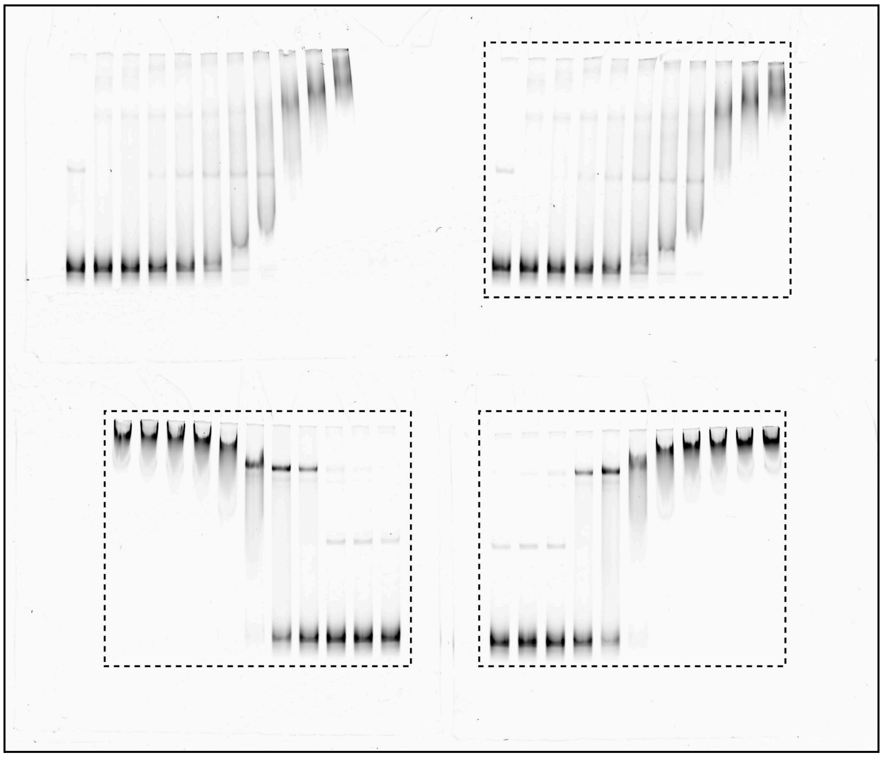

mismatch 13-17; left panel

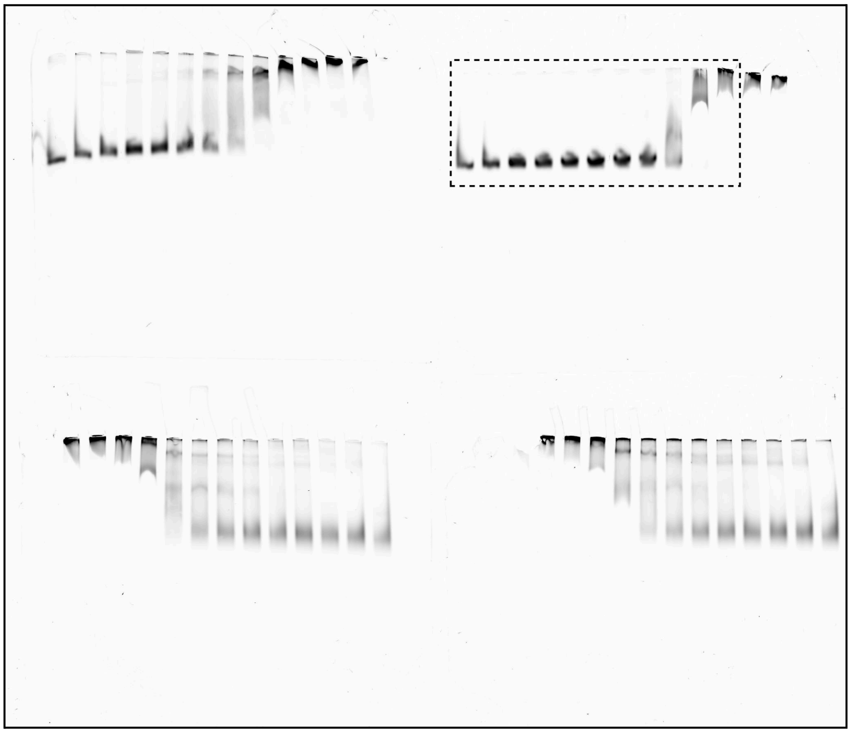

mismatch 13-17; center panel

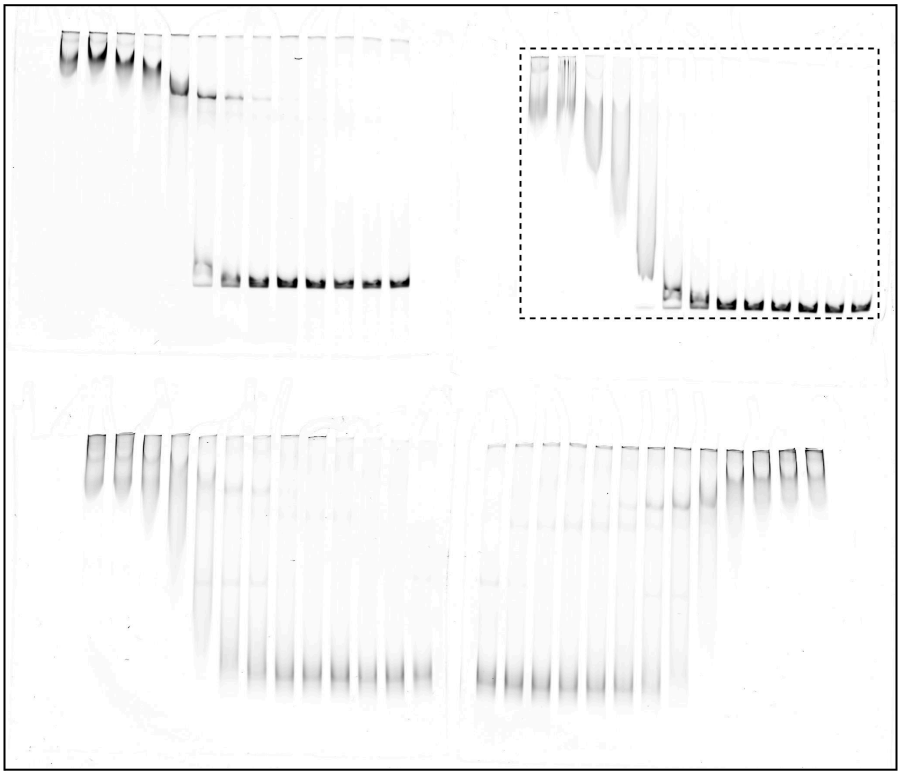

mismatch 13-17; right panel

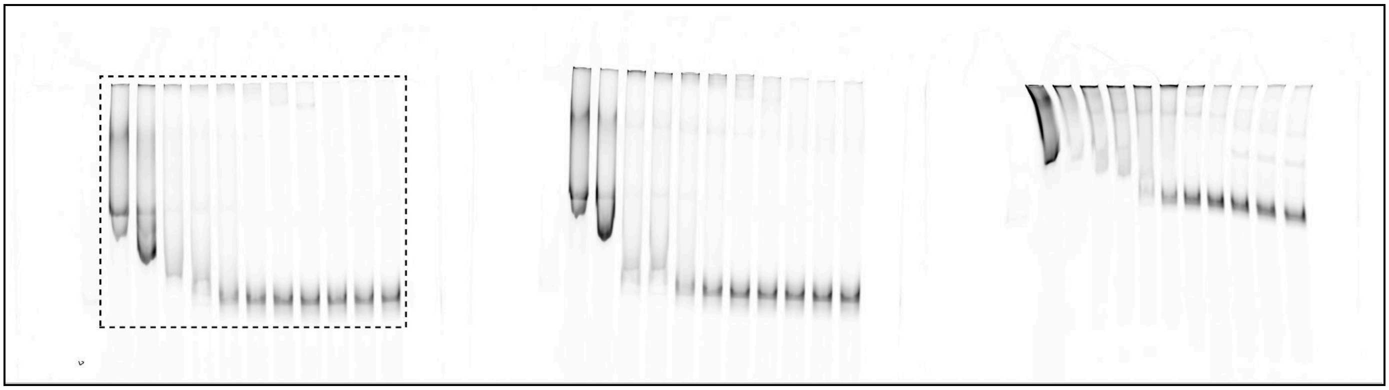

mismatch 19-23; left panel

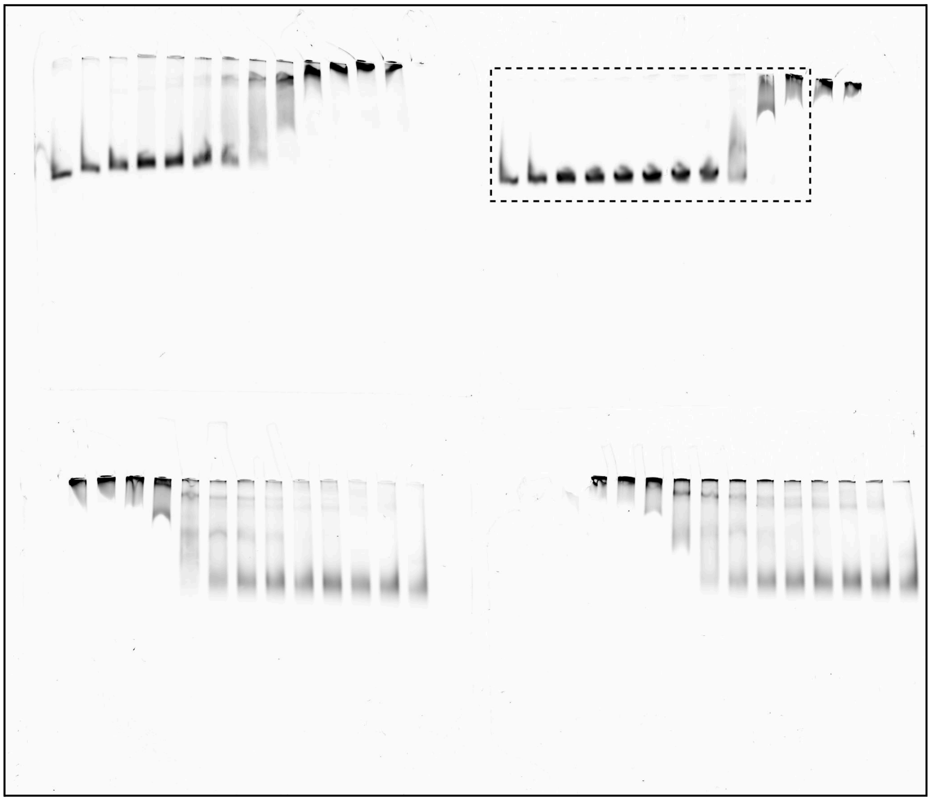

mismatch 19-23; center panel

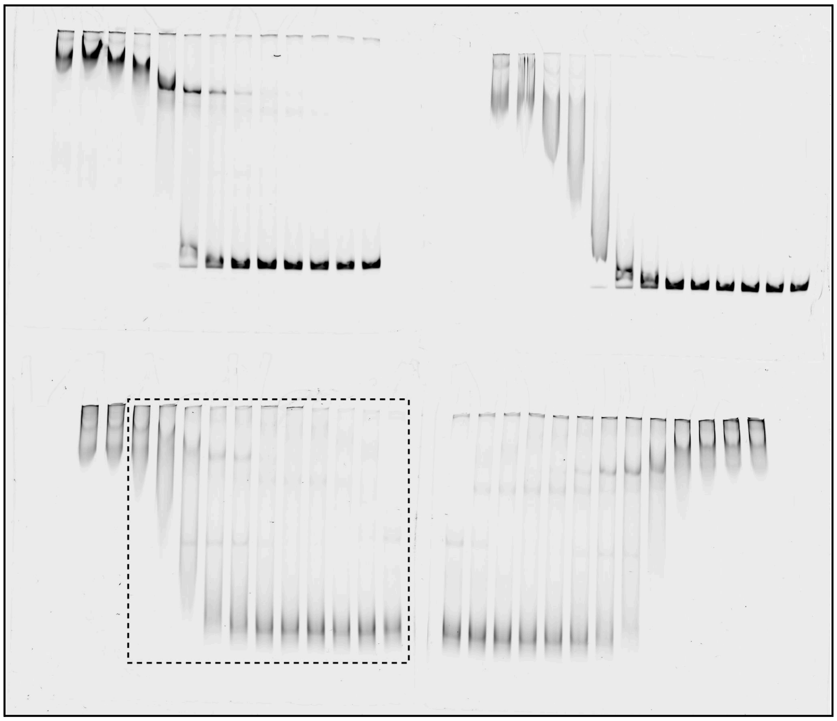

mismatch 19-23; right panel

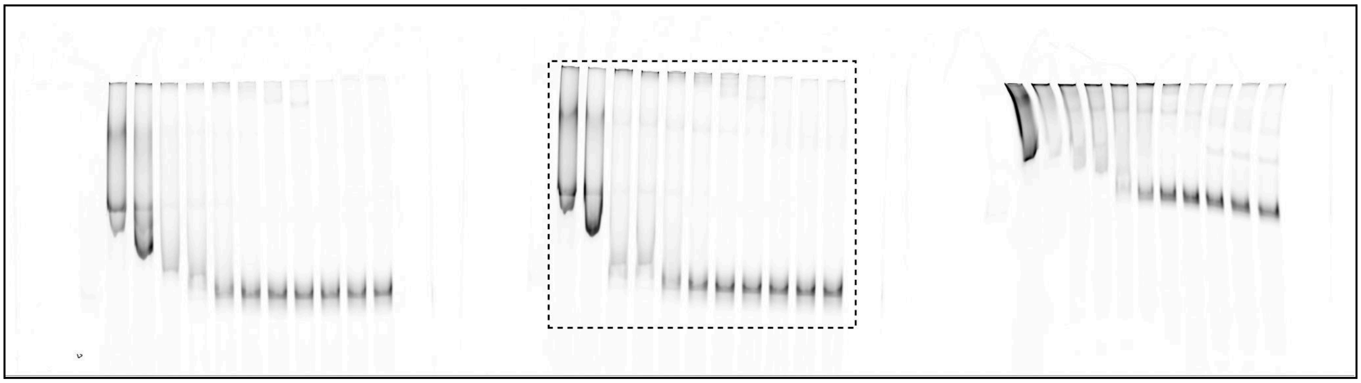

**Supplementary Fig. 11**

no mismatch; left panel

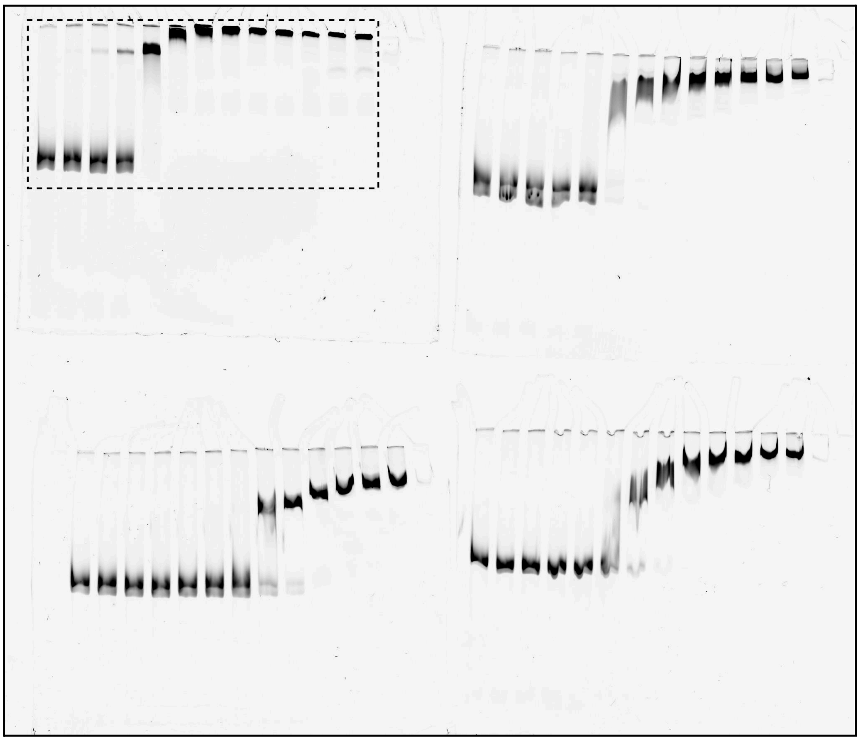

no mismatch; center panel

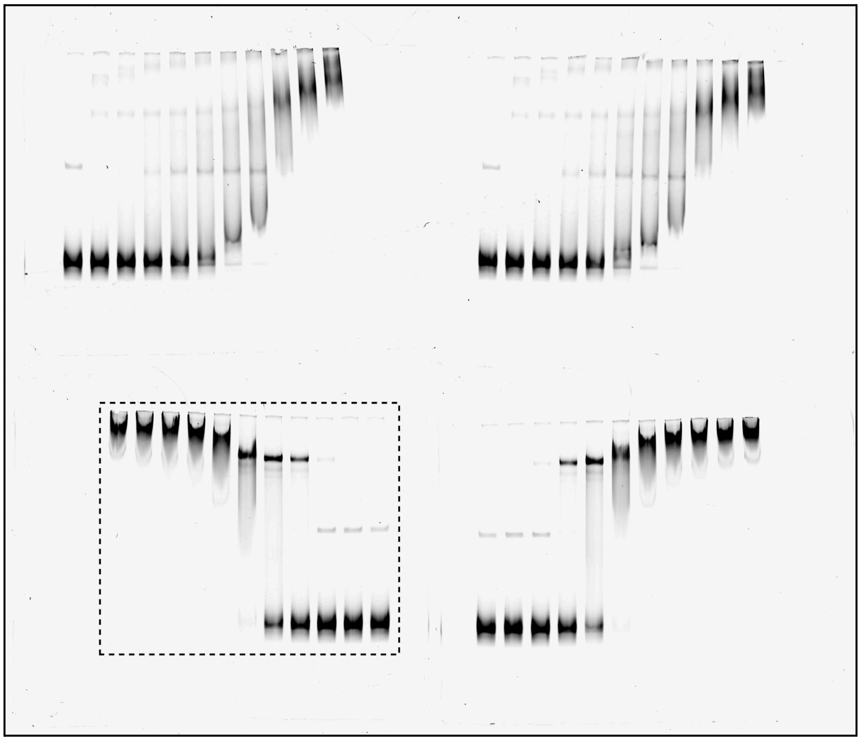

no mismatch; right panel

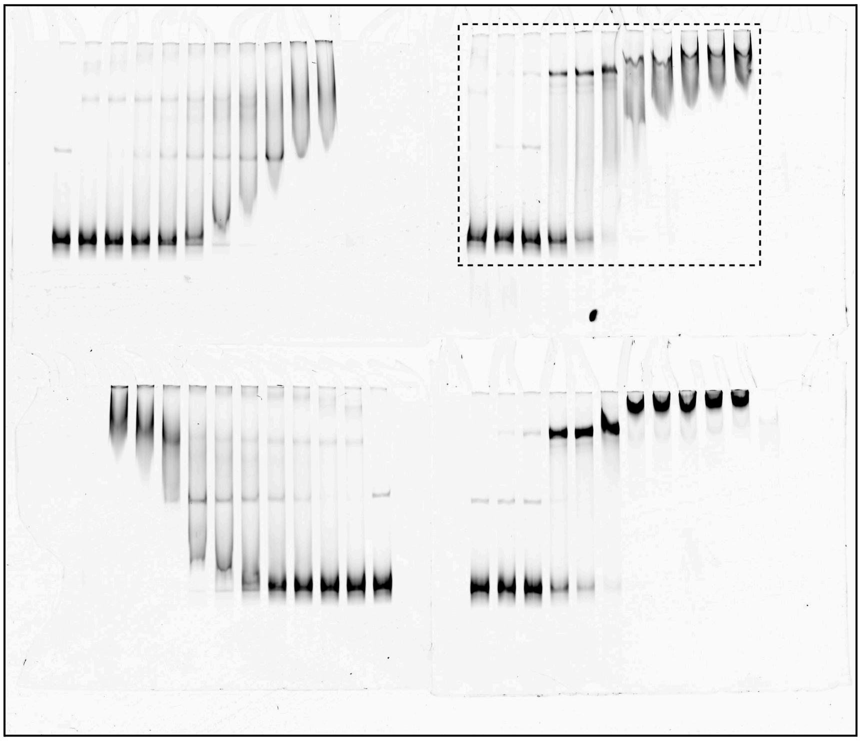

mismatch 13-17; left panel

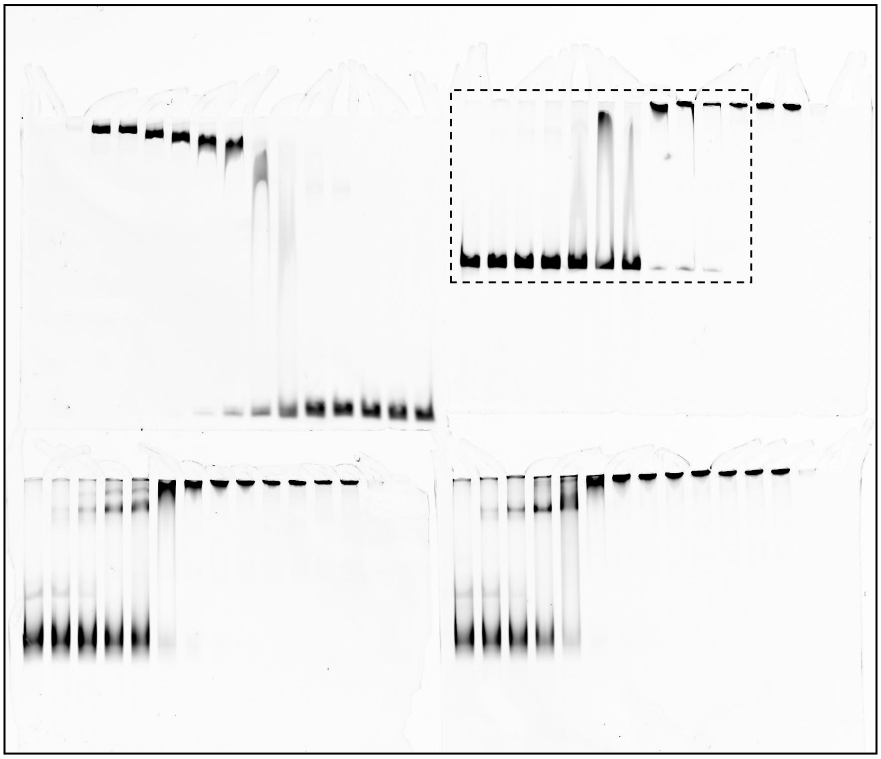

mismatch 13-17; center panel

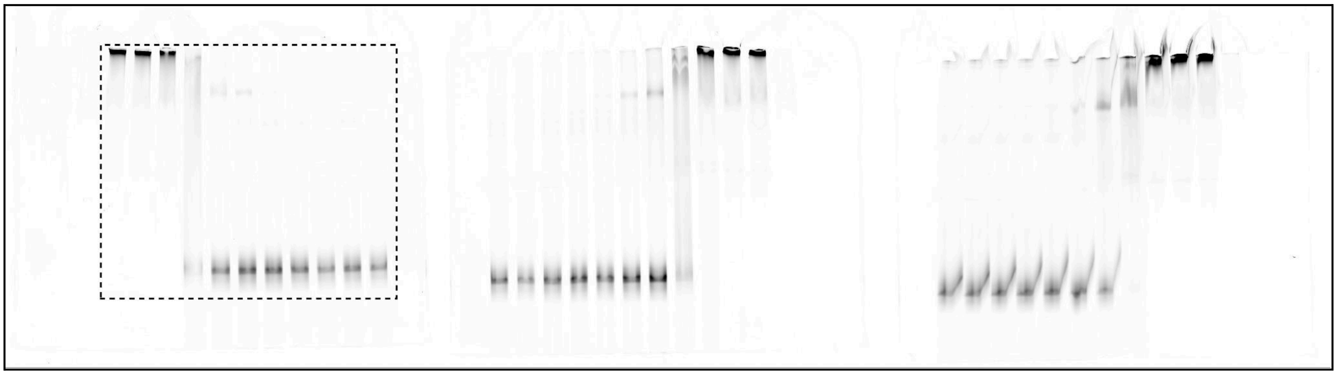

mismatch 13-17; right panel

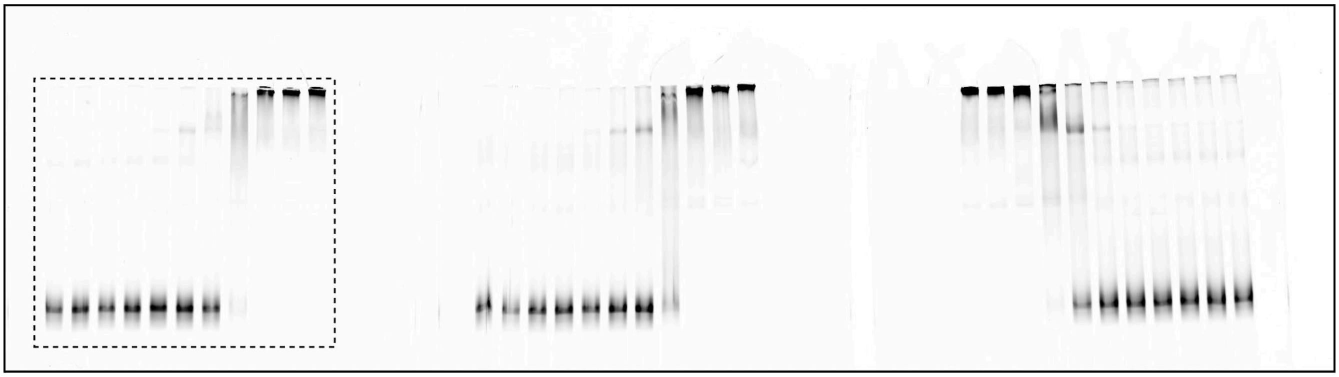

mismatch 19-23; left panel

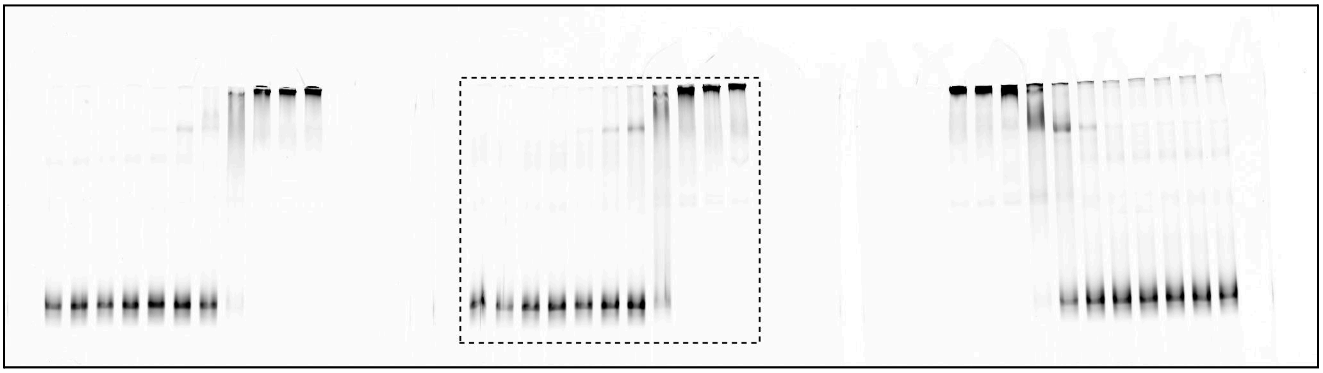

mismatch 19-23; center and right panel

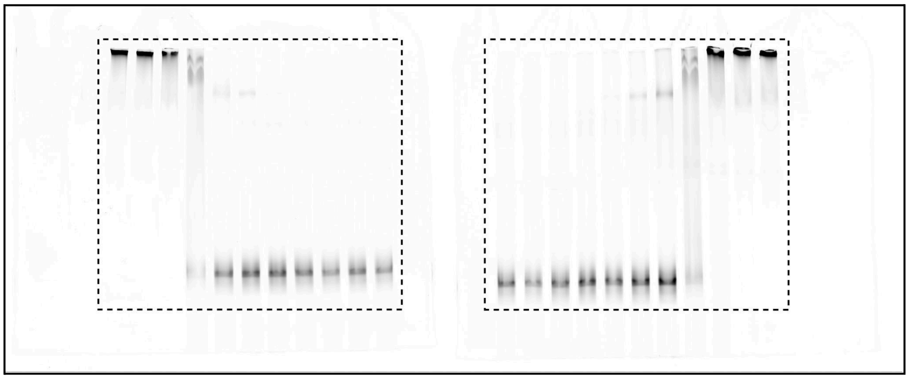

mismatch 25-29; left panel

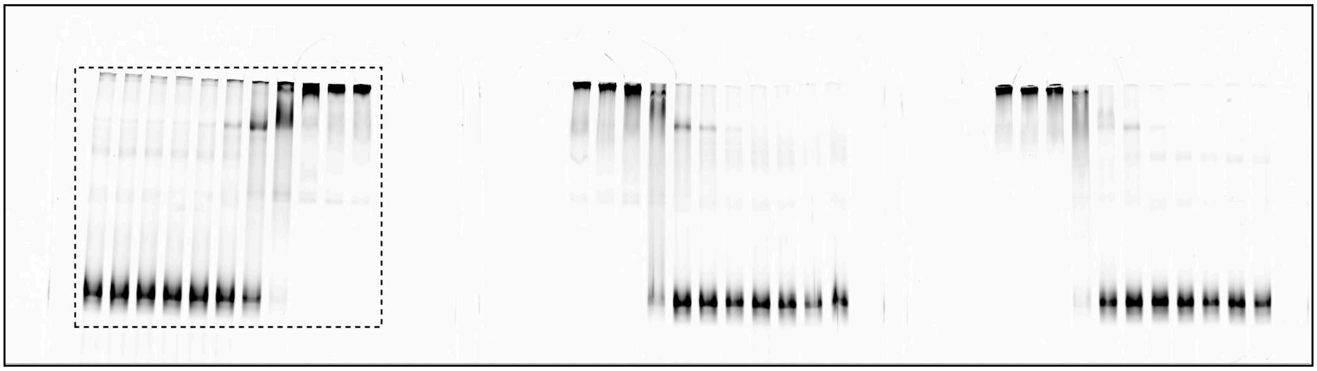

mismatch 25-29; center panel

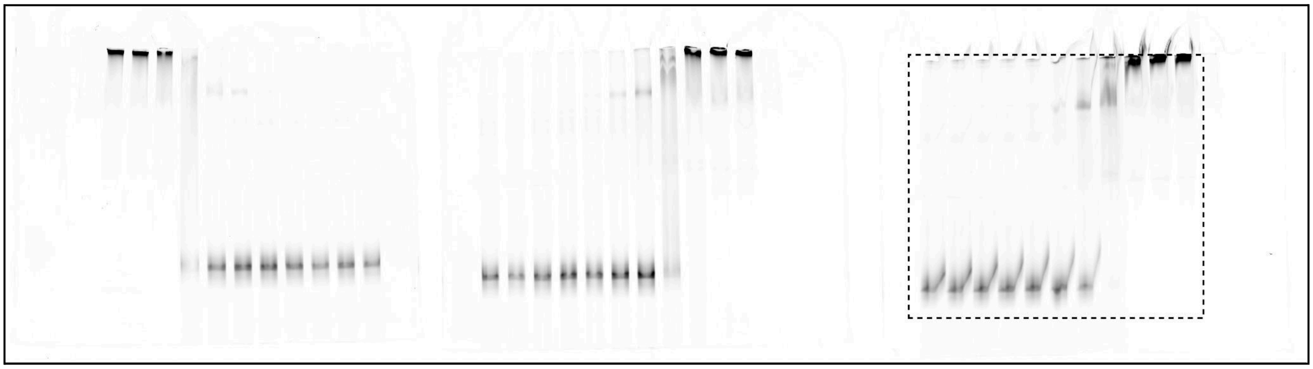

mismatch 25-29; right panel

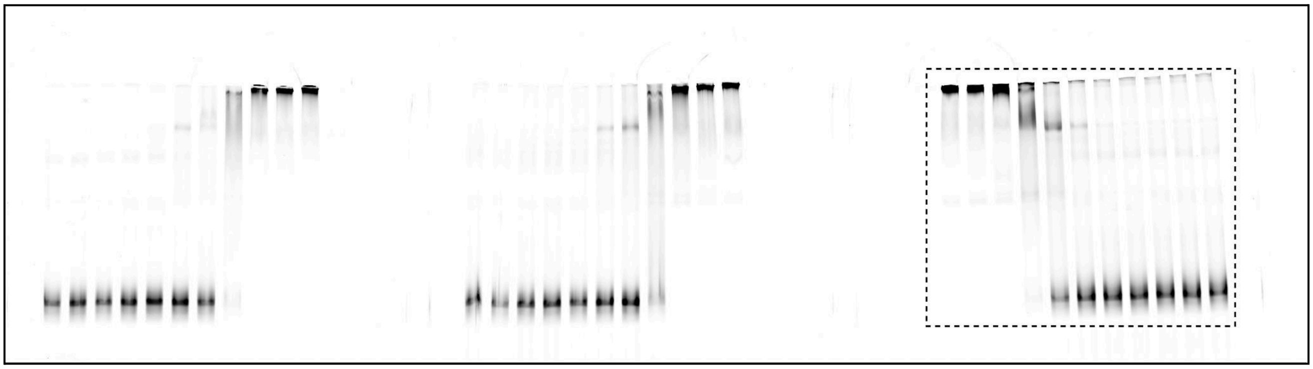

mismatch 31-32; left, center and right panel

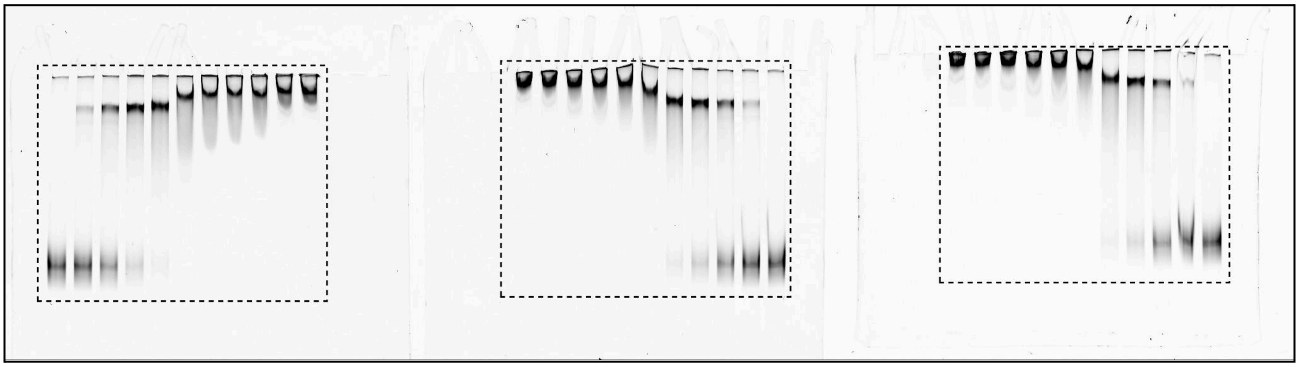

**Supplementary Fig. 12a**

Cas7Δ191-208 (GSSG); left panel

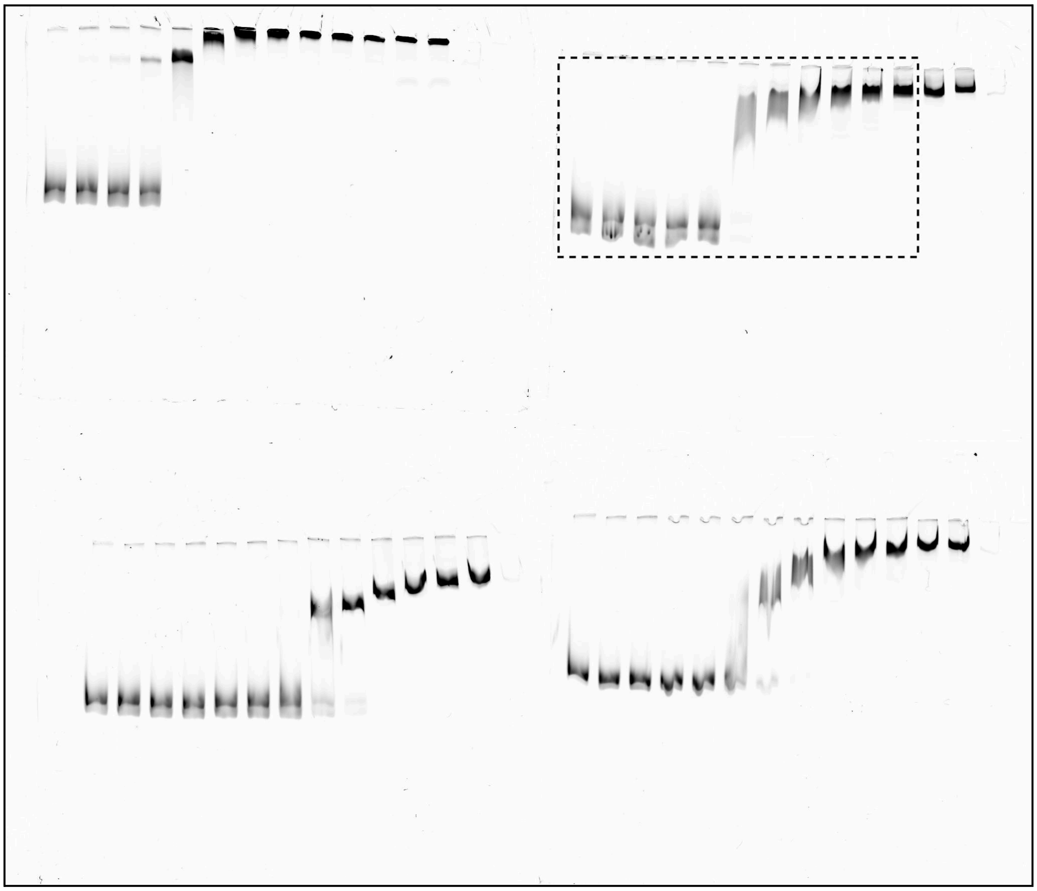

Cas7Δ191-208 (GSSG); center panel

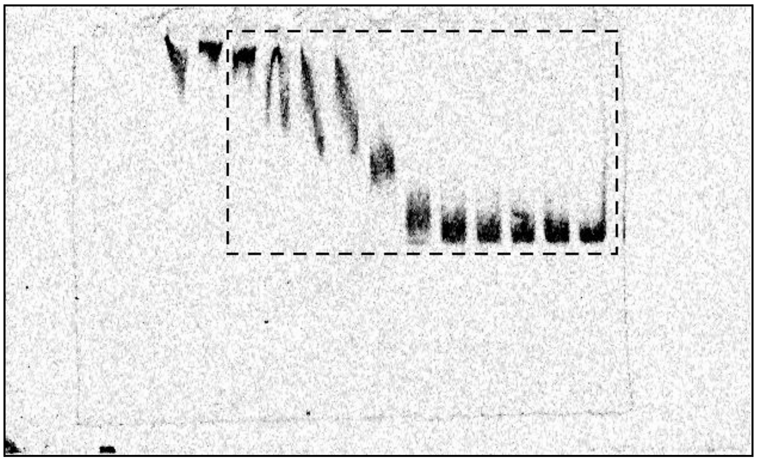

Cas7Δ191-208 (GSSG); right panel

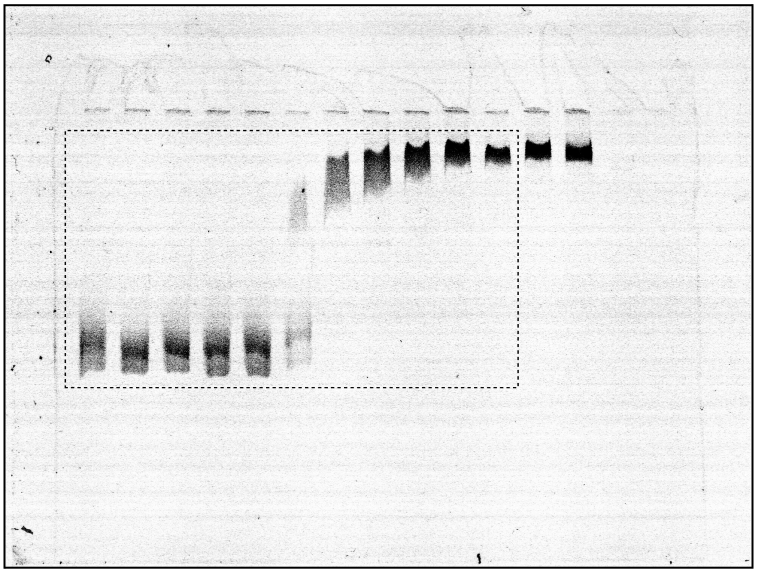

Cas7Δ184-216 (GSSG); left panel

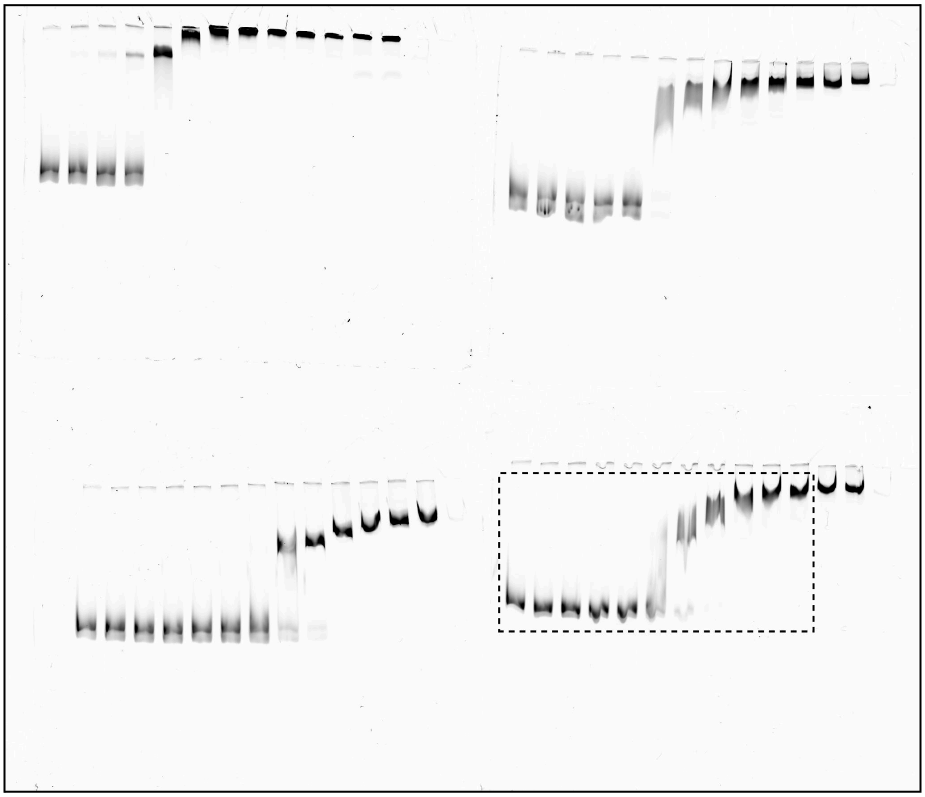

Cas7Δ184-216 (GSSG); center panel

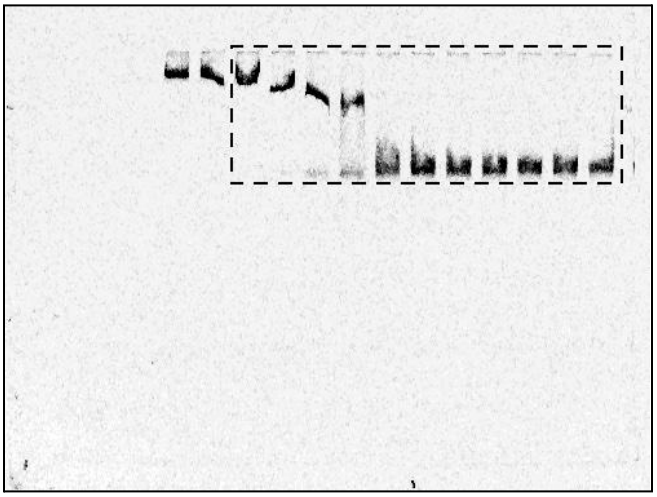

Cas7Δ184-216 (GSSG); right panel

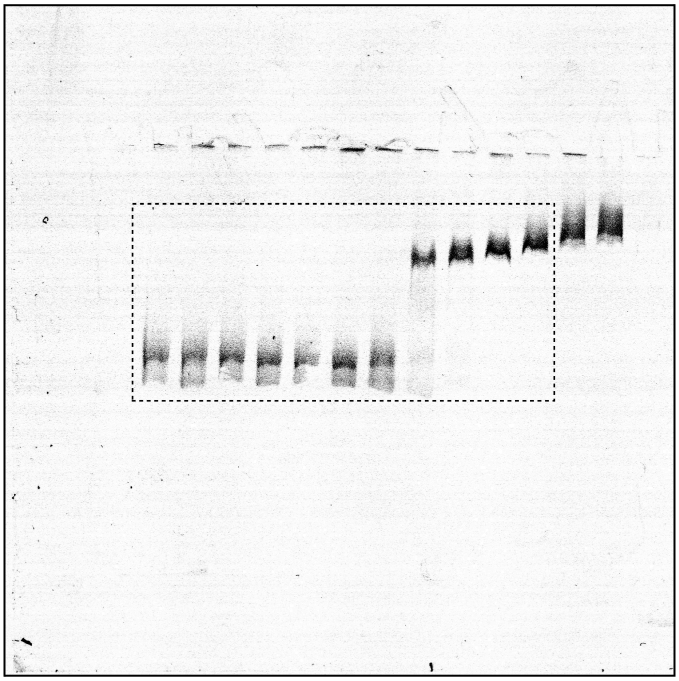

Cas7Δ177-224 (GSSG); left panel

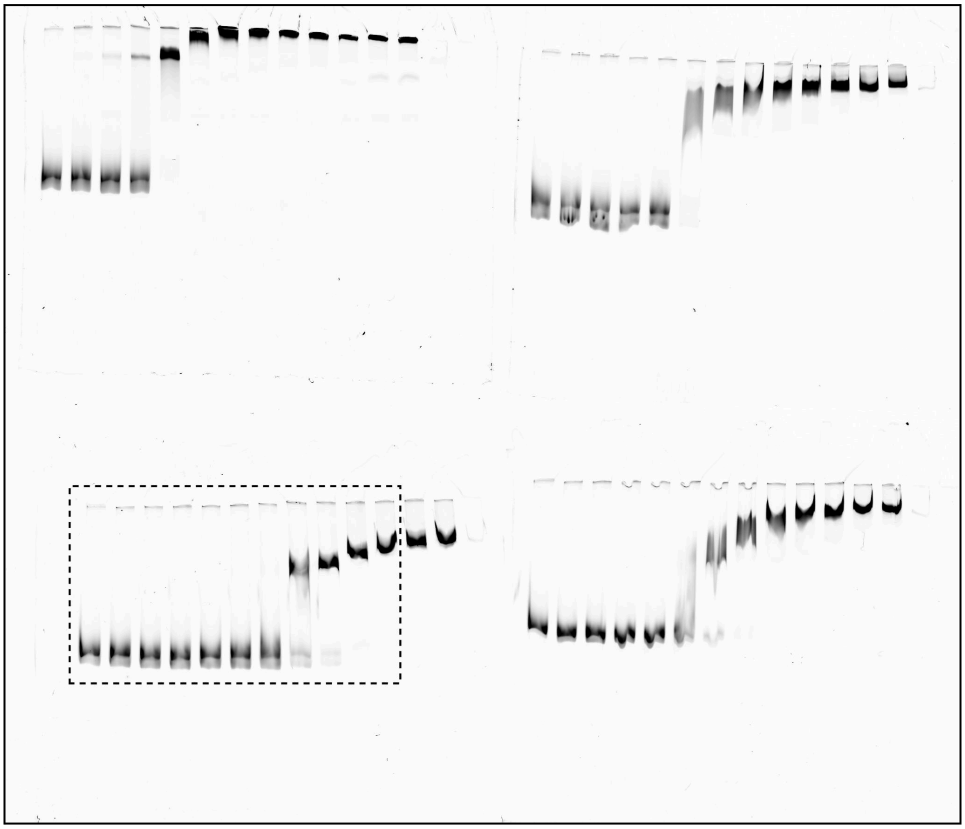

Cas7Δ177-224 (GSSG); center panel

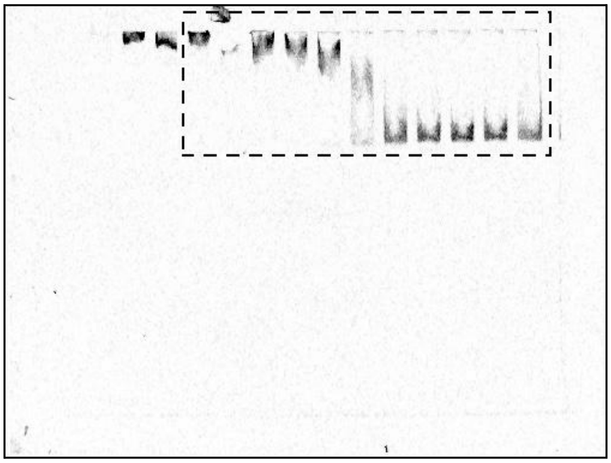

Cas7Δ177-224 (GSSG); left panel

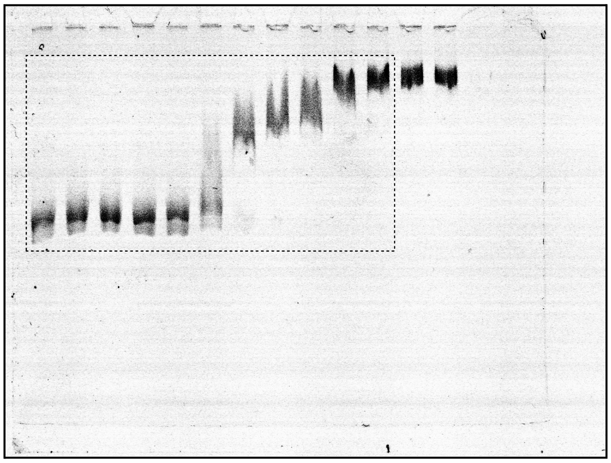

**Supplementary Fig. 12b**

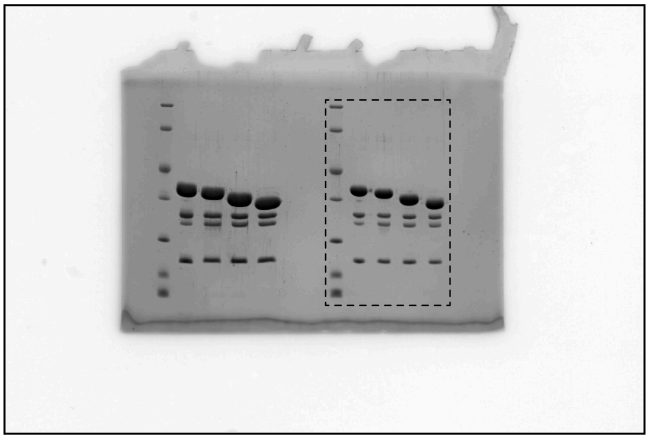

**Supplementary Fig. 13a**

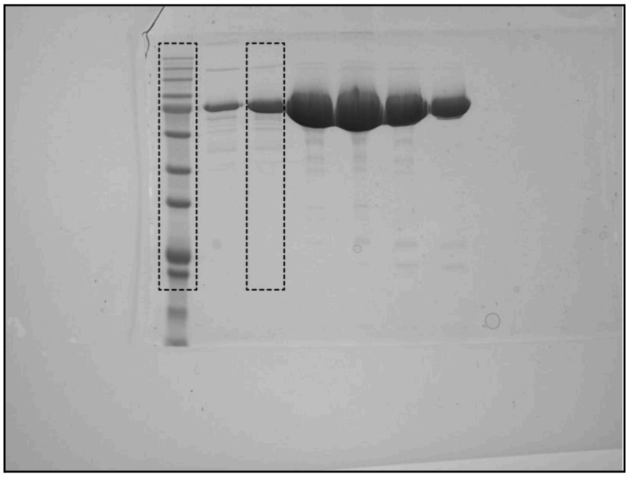

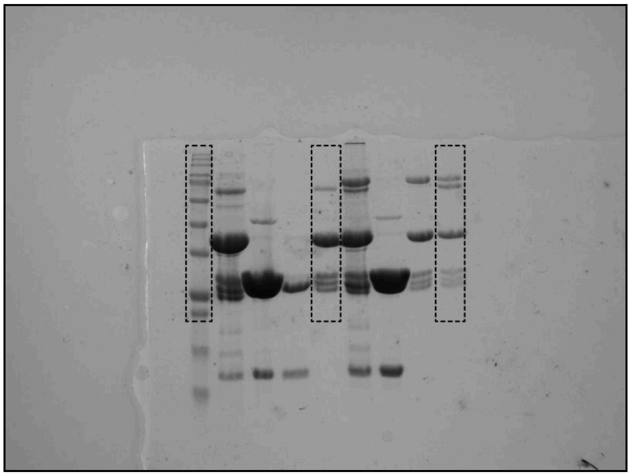

**Supplementary Fig. 13b**

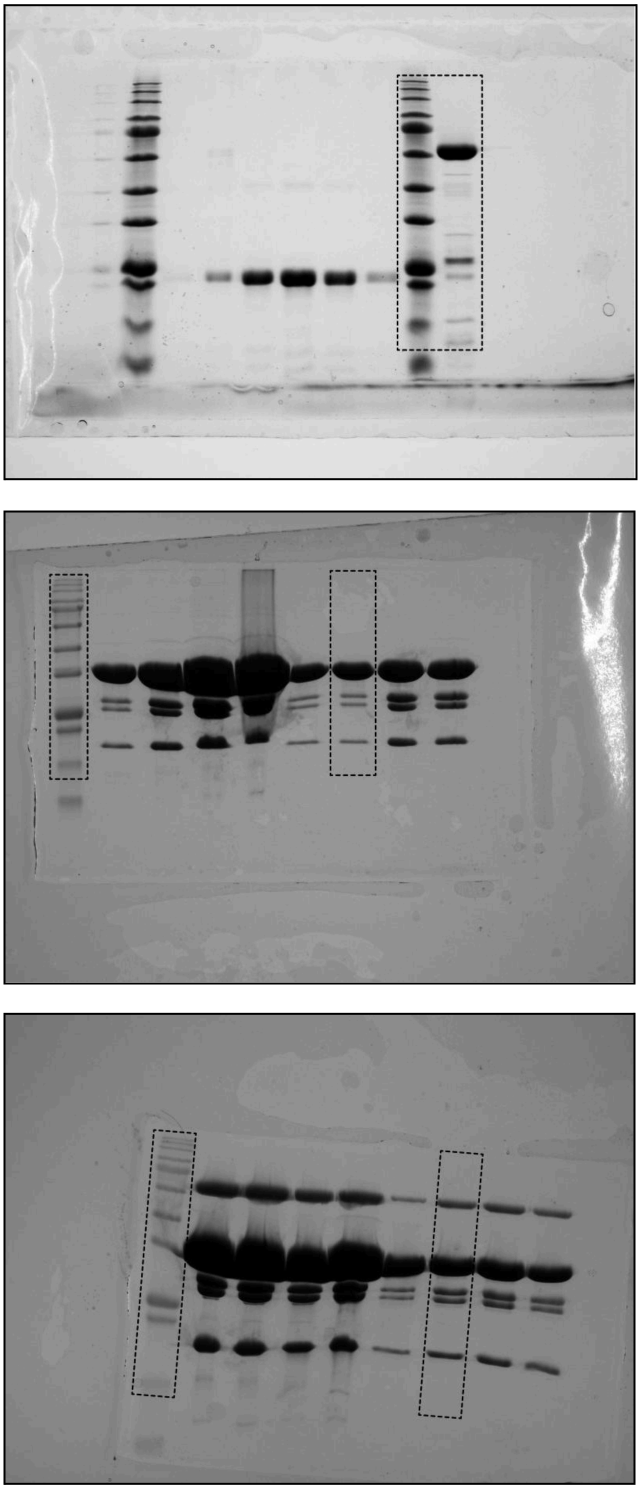

**Supplementary Fig. 16a**

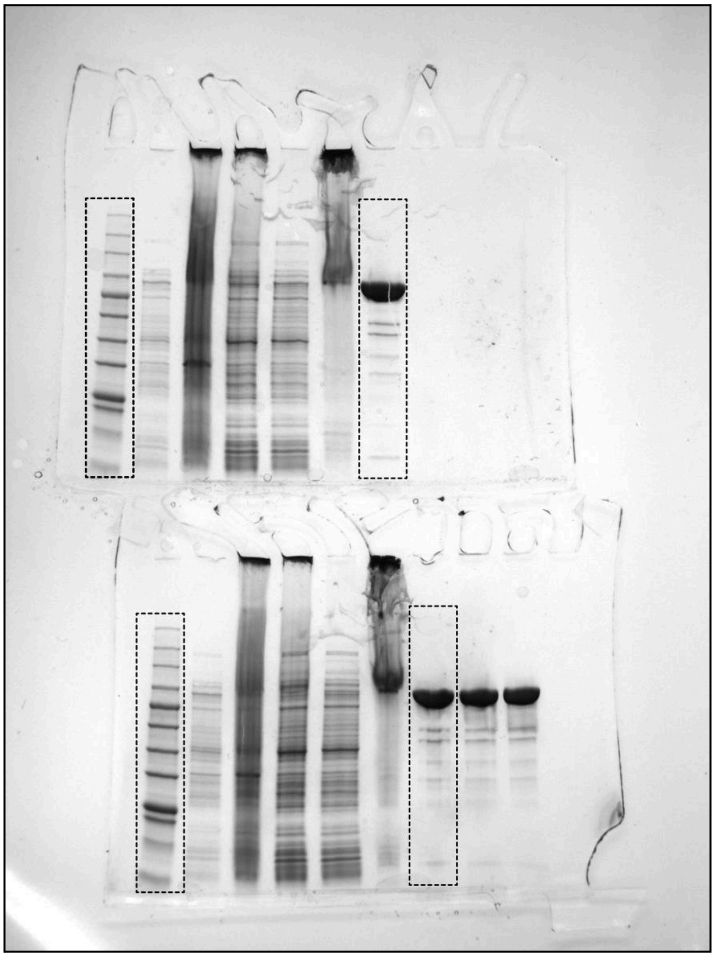

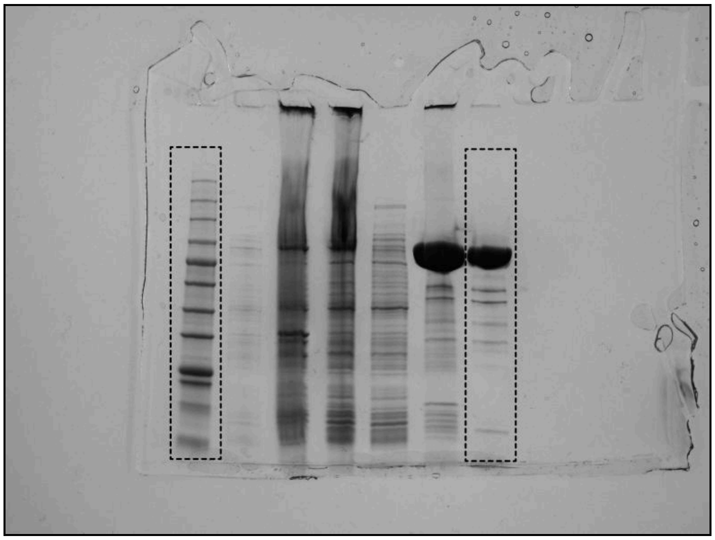

**Supplementary Fig. 16b**

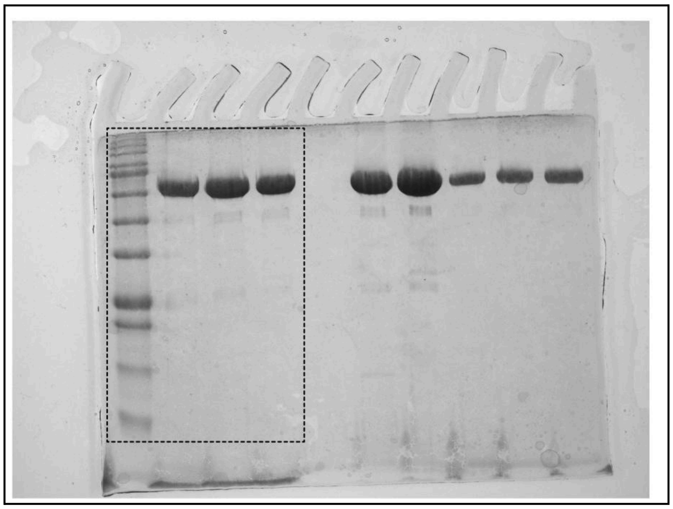

Supplement: Supplementary file 1 — Supplementary Information [file 41467_2024_53778_MOESM1_ESM.pdf]
