# Supplementary material for: Structural variation of types IV-A1- and IV-A3-mediated CRISPR interference
Source: Nat Commun. 2024 Oct 29;15:9306. doi: 10.1038/s41467-024-53778-1 (PMC11519345; doi:10.1038/s41467-024-53778-1)
Supplement: Supplementary file 3 — Description of Additional Supplementary Files [file 41467_2024_53778_MOESM3_ESM.pdf]

Supplementary Data 1.
Commercial reagents. List of commercial reagents used in this study.

Supplementary Data 2.
Plasmid Sequences. List and sequences of plasmids used in this study.
